# The importance of public health, poverty reduction programs and women’s empowerment in the reduction of child stunting in rural areas of Moramanga and Morondava, Madagascar

**DOI:** 10.1371/journal.pone.0186493

**Published:** 2017-10-18

**Authors:** Chitale Remonja Rabaoarisoa, Rado Rakotoarison, Nivo Heritiana Rakotonirainy, Reziky Tiandraza Mangahasimbola, Alain Berthin Randrianarisoa, Ronan Jambou, Inès Vigan-Womas, Patrice Piola, Rindra Vatosoa Randremanana

**Affiliations:** 1 Epidemiology unit, Institut Pasteur de Madagascar, Antananarivo, Madagascar; 2 Immunology of Infectious Diseases unit, Institut Pasteur de Madagascar, Antananarivo, Madagascar; 3 Department of Parasitology- Mycology, Institut Pasteur de Côte d’Ivoire, Abidjan, Côte d’Ivoire; 4 Epidemiology and Public Health unit, Institut Pasteur du Cambodge, Phnom Penh, Cambodge; Centre Hospitalier Universitaire Vaudois, FRANCE

## Abstract

**Background:**

Malnutrition accounts for 45% of mortality in children under five years old, despite a global mobilization against chronic malnutrition. In Madagascar, the most recent data show that the prevalence of stunting in children under five years old is still around 47.4%. This study aimed to identify the determinants of stunting in children in rural areas of Moramanga and Morondava districts to target the main areas for intervention.

**Methods:**

A case-control study was conducted in children aged from 6 to 59.9 months, in 2014–2015. We measured the height and weight of mothers and children and collected data on child, mother and household characteristics. One stool specimen was collected from each child for intestinal parasite identification. We used a multivariate logistic regression model to identify the determinants of stunting using backwards stepwise methods.

**Results:**

We included 894 and 932 children in Moramanga and in Morondava respectively. Stunting was highly prevalent in both areas, being 52.8% and 40.0% for Moramanga and Morondava, respectively. Stunting was most associated with a specific age period (12mo to 35mo) in the two study sites. Infection with *Trichuris trichiura* (aOR: 2.4, 95% CI: 1.1–5.3) and those belonging to poorer households (aOR: 2.3, 95% CI: 1.6–3.4) were the major risk factors in Moramanga. In Morondava, children whose mother had activities outside the household (aOR: 1.7, 95% CI: 1.2–2.5) and those perceived to be small at birth (aOR: 1.6, 95% CI: 1.1–2.1) were more likely to be stunted, whereas adequate birth spacing (≥24months) appeared protective (aOR: 0.4, 95% CI: 0.3–0.7).

**Conclusion:**

Interventions that could improve children’s growth in these two areas include poverty reduction, women’s empowerment, public health programmes focusing on WASH and increasing acceptability, and increased coverage and quality of child/maternal health services.

## Introduction

The World Health Organization has called for global action to reduce child stunting by 40% by 2025 [1], and there is currently a global mobilization effort against chronic malnutrition. Malnutrition accounts for 45% of the mortality in children under 5 in 2011 [2], and recent estimates in 2015 showed that nutritional deficiencies caused 405 700 deaths [3]. Child growth is influenced by several factors including macro- and micronutrient deficiencies, food insecurity, infections from inadequate access to water, sanitation and hygiene, lack of access to health services and poor socio-economic conditions[4]. Chronic malnutrition has long-lasting consequences on educability, future work capacity, income-earning ability and susceptibility to obesity and chronic diseases [5–8].

Stunting is the most common manifestation of chronic malnutrition and refers to a child who is short for his/her age. Wasting, also known as acute malnutrition refers to low weight for height when a child is thin for his/her height. In low-income countries, 37.6% of children under 5 years were stunted in 2014 [9]. Madagascar is a very low-income country (gross national annual income per capita of 420 USD) [10], and the latest data show that the prevalence of stunting among children aged less than 5 years is still approximately 47.4% [11]. Madagascar is among the countries with the highest prevalence of stunting [12], and this prevalence is even higher in the Central Highlands regions, reaching over 60% [11]. In addition, infectious diseases remain the main causes of death in children under 5, with respiratory infections being the first cause (18.0%) and diarrheal diseases the fourth (9.0%)[13]. The National Office of Nutrition (ONN) of Madagascar initiated a large-scale community-based nutrition intervention in 1999 that included monthly growth-monitoring activities for infants and young children, cooking demonstrations, community mobilization, and nutrition and hygiene education for primary caregivers [14]. The evaluation of the long-term effects of the existing program showed minor significant effects in young children on weight-for-age but no effects on height-for-age [15].

Comprehensive knowledge about the determinants of stunting in local contexts is necessary to reduce the prevalence of stunting by improving and strengthening nutrition intervention programs. A secondary analysis of the most recent Demographic and Health Survey (2009) data suggested a disparity between regions of Madagascar in terms of the odds of stunting among children [16]. We conducted this current study in rural areas of the Moramanga and Morondava districts, located in the Alaotra-Mangoro and Menabe regions respectively. These 2 study areas have different nutritional profiles; Alaotra-Mangoro region is located in an area with high stunting prevalence (50–60%), and Menabe region is in an area with average stunting prevalence (30–40%) [11]. The area of highest prevalence of stunting in Madagascar (≥60%) is in the central highland.

The primary objective was to identify the main determinants of stunting among children aged 6 to 59.9 months in the two districts to further understand which actions could be implemented to accelerate stunting reduction.

## Methods

### Study areas

We conducted this study in two rural areas of Madagascar: the Health and Demographic Surveillance Site (HDSS) of Moramanga and the municipality of Bemanonga, district of Morondava. The two selected areas belong to 2 regions with different nutritional profiles: Alaotra-Mangoro region is an area with high stunting prevalence whereas Menabe region is located in an area with average stunting prevalence. We assumed these differences could be related to regional differences in stunting determinants. The HDSS of Moramanga is located in the central eastern part of Madagascar and is composed of three municipalities, 30 villages and 60,000 inhabitants. These three municipalities have a total of 9 operational health facilities (1 hospital, 8 health centers). The municipality of Bemanonga is located in the southwestern part of Madagascar. This study was implemented in 13 of the 34 villages in Bemanonga. The 13 villages had an estimated total population of 13,700 and had two health centers.

### Study design

The participants of this community-based case-control study were children aged 6 to 59.9 months and their mothers or caregivers.

#### Sample size

Sample size was calculated to detect an odds ratio of 1.5 for the association between stunting and intestinal parasite carriage, with a 95% confidence interval, a statistical power of 80% and an expected prevalence of parasitic infection of 10% in non-malnourished children. The total sample size was 1620 children in both study areas (405 cases and 405 controls at each study site).

#### Sampling

Firstly, a screening phase was conducted by measuring weight, height/length of all children under 5 years. A simple random sampling of non-malnourished and stunted children was later performed to form the case and control groups. Cases were children aged 6–59.9 months with stunting, defined as a height-for-age z-score<-2SD of the median height of the WHO reference population. Controls were children aged 6–59.9 months without stunting nor wasting. If mothers had more than one eligible child, only the first randomly selected child was considered.

Children under 5 years with disabilities preventing anthropometric measurements were not included in the screening phase. Those with both stunting and wasting and those not accompanied by their own mothers or caregivers during the survey were not included in the case-control study.

#### Data collection

Locally recruited fieldworkers were trained to perform the anthropometric measurements to identify the source populations of cases and controls and to conduct interviews with the mothers. Data were collected from January to July 2014 in Moramanga and from August to November 2014 in Morondava. All eligible children were measured during the screening phase in both sites. Weight was measured to the nearest 100 g using a hanging scale (Salter^®^) for children weighing ≤ 25 kg and with a weighing scale (Gima^®^) for those weighing > 25 kg, those for whom using a hanging scale was difficult (agitated, did not want to be separated from their mothers, etc.) and for mothers.

Length/height was measured to the nearest 0.1 cm in a recumbent position for children < 24 months and in a standing position for those who were older using collapsible length/height boards. All scales were frequently calibrated, before and during the survey.

We interviewed mothers/caretakers in Malagasy using a structured questionnaire. The following information was collected: maternal characteristics (education, occupation, dietary patterns, pregnancy history, antenatal information, marital status), household characteristics (housing characteristics, water source, sanitation, goods, possession of land and livestock, feeding practices) and child characteristics (date of birth, sex, childhood illness symptoms two weeks before the survey, immunization status, child diet, place of delivery, vitamin A capsule intake and use of de-worming medication within 6 months before the survey). The information collected on child diet were breastfeeding status, age at introduction of supplementary food and all food/beverages consumed by the child the day before the survey.

#### Parasitological analysis

For each randomly selected child, we collected approximately 10 g of fresh stool sample for parasite identification. Intestinal parasites, vegetative forms, cysts and eggs were detected by direct examination of smear on a clean glass slide after dilution of approximately 2 mg of stool in isotonic salt solution. Each slide was subsequently stained with Lugol’s Iodine solution and examined by conventional light microscopy by 2 certified laboratory technicians. Stool samples were tested for opportunistic parasites at the Institut Pasteur de Madagascar. Opportunistic parasites are parasites that take advantage of an opportunity not normally available such as host with a weakened immune system or an altered microbiota to infect the host. For this purpose, stool samples were conserved at room temperature in a 2.5% potassium dichromate solution until use. Microscopic diagnosis of oocysts of *Cryptosporidium spp*., *Isospora belli* and *Cyclospora cayetanensis* was performed using the modified Ziehl-Neelsen acid fast stain technique [17]. *Microsporidia* were detected by Trichrome stain [18,19]. For opportunistic parasites, diagnosis was confirmed by molecular analysis of positive samples detected by microscopy. DNA extraction from stool samples was performed using a modified QIAamp^®^ DNA Stool Mini Kit protocol [20]. Briefly, before extraction, 200 μg of stool samples, conserved in potassium dichromate, were washed twice with PBS 1x (Phosphate buffer saline, Invitrogen). A sonication step, 30 minutes at 4°C, was subsequently performed, and 1.4 ml of ASL lysis buffer was added to the pretreated stool. This step was followed by incubating the preparation for 5 min at 95°C prior to DNA extraction, as recommended by the manufacturers. *Cryptosporidium parvum*, *Entamoeba histolytica* and *Giardia lamblia* were simultaneously detected by real time qPCR multiplex assay TaqMan StepOne-plus using primers and TaqMan probes following the protocol already described by Verweij *et al* [21,22]. *Microsporidia* species, i.e., *Enterocytozoon bieneusi* and *Encephalitozoon spp*., were also detected by real time qPCR assay as described [21].

#### Data management and statistical analysis

Data were collected on electronic forms using a Microsoft Access database. We examined anthropometric data for completeness and plausibility using Emergency Nutrition Assessment software (ENA for Smart, Centers for Disease Control and Prevention). Weight and length/height of the children were converted into height-for-age z-scores and weight-for-height z-scores according to the 2006 WHO child growth standards [23] and we used R software version 3.0 for all subsequent analyses. After cleaning the data to address inconsistencies, descriptive analyses were performed.

A wealth index was created using variables related to ownership of selected household assets, housing materials (floor, wall, and roof materials), access to utilities (electricity, safe water, latrine, bathroom, cooking location), family size and combustible used for cooking. We used principal component analysis for continuous variables and multiple correspondence analyses for categorical variables. We subsequently grouped the wealth index score into quartiles, with Quartile 1 representing the poorest segment of the population and Quartile 4, the wealthiest.

Food consumed by children the day before the survey was used to calculate the dietary diversity score which is defined as the number of food groups consumed by the child the previous day. We considered seven food groups: (1) cereals, roots and tubers; (2) legumes and nuts; (3) milk and its derivatives; (4) meat products (meat, poultry, offal, and fish); (5) eggs; (6) fruits and vegetables; and (7) oils and fats. Low dietary diversity score was defined as consumption of ≤3 food groups for children < 36 mo and ≤4 food groups for children≥36 mo.

The Body Mass Index (BMI) for mother was assessed by dividing the weight (kilogram) by the square of body height (meter); we used three categories for the analyses “thin”: BMI<18.5 kg/m^2^, “normal”: BMI between 18.5 and 25 kg/m^2^ and “overweight”: BMI>25 kg/m^2^.

Household food consumption score was constructed according to recommendations from the World Food Program [24]. Six groups of foods were considered, including (1) cereals and tubers, (2) legumes and peas, (3) vegetables, (4) fruits, (5)meat-based foods, and (6) milk and its derivatives (data on the consumption of sugar, oil and condiments were not available). The calculation of the score was performed with the consumption frequency of these food groups the week before the survey and a standard weight according to the groups of the consumed foods. The obtained score was then classified into 3 categories: poor, limited and acceptable.

We assessed candidate variables for the multivariate analysis using bivariate analysis; all explanatory variables with a p-value<0.2 were included in a backward stepwise logistic regression analysis. Odds ratios and 95% confidence intervals (95% CIs) were calculated to estimate the strength of the association between the occurrence of stunting and the potential explanatory variables. Interactions between explanatory variables were checked.

We did not perform imputation of missing data, and in Morondava, we included observations with missing values as additional categories.

#### Ethical consideration

Parents or children’s guardians were informed about the study and signed a letter of consent before inclusion. Children with severe acute malnutrition were referred to the nearest health center; those with any identified parasites were treated according to the national treatment guidelines. The protocol of the study was approved by the National Ethics Committee of the Ministry of public health of Madagascar (042-MSANP/CE, June 13^th^ 2014).

## Results

### Participant characteristics

**Fig 1:**
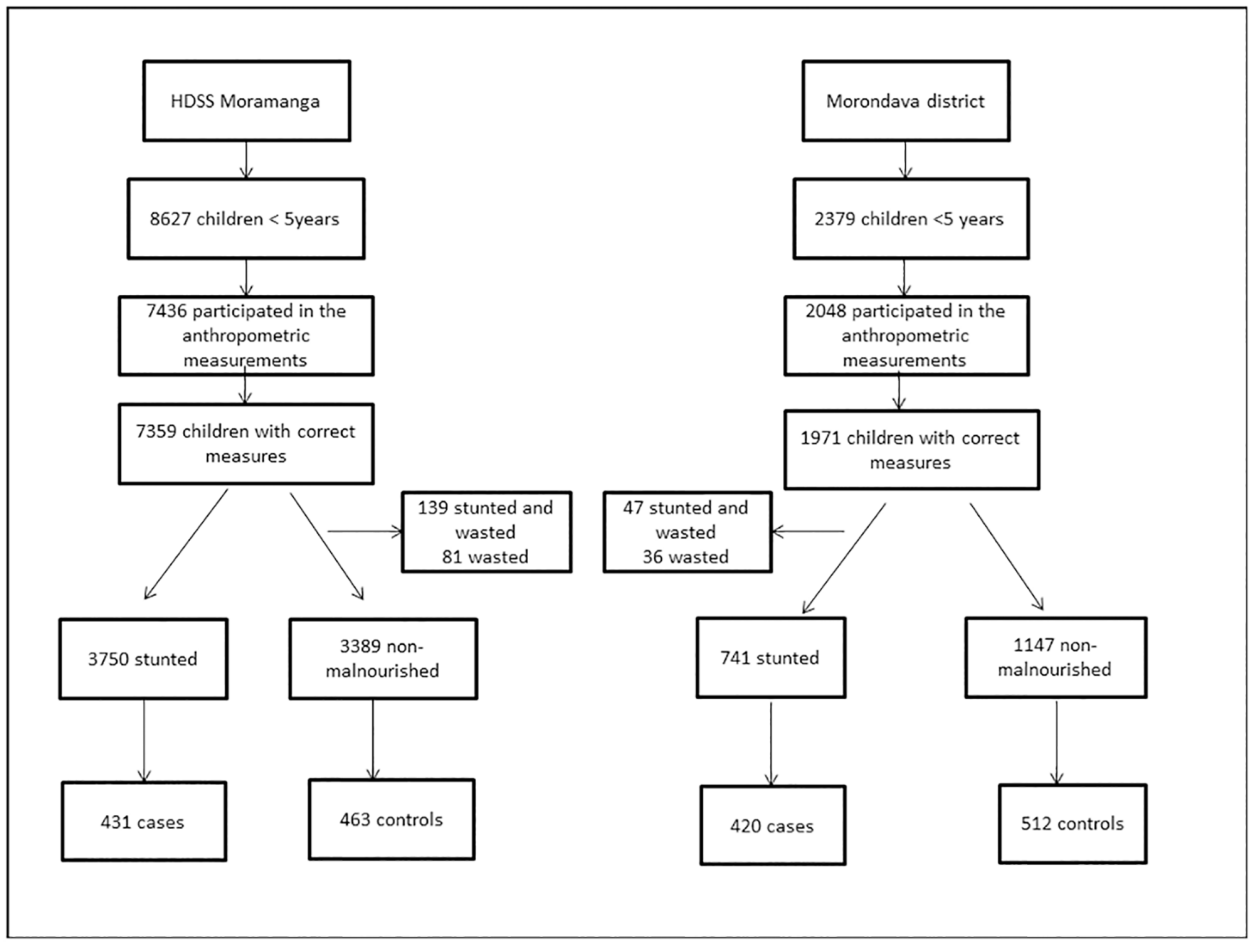
Flowchart describing the recruitments of participants. 1a.Name of the 2 study areas, 1b.Number of children under five years in the 2 study areas, 1c. Number of children who participated in the anthropometric measurements in the 2 study areas, 1d. Number of children who participated in the anthropometric measurements and with valid measures in the 2 study areas, 1e. Number of excluded children among those with valid measures in the 2 study areas 1f. Number of stunted and non-malnourished children in the 2 study areas, 1g. Number of stunted and non-malnourished children randomly selected and included in the case-control study in the 2 study areas.

After random sampling, 894 children (431 cases and 463 controls) were included in the case-control study in Moramanga and 932 children (420 cases and 512 controls) were included in Morondava. In Moramanga, 7436 children under 5 years participated in the anthropometric measurements, of which 7359 (99.0%) had valid measures. For the case-control study, we randomly selected 7139 children without wasting (3750 stunted and 3389 non-malnourished). In Morondava, 2048 children were screened, of which 1971 (96.2%) had valid measurements and 1888 (1147 non-malnourished and 741 stunted) were randomly selected for the case-control study. The prevalence of chronic malnutrition was 52.8% (95% CI 51.7–54.0) and 40.0% (95% CI: 37.8–42.1) in Moramanga and Morondava, respectively. The prevalence of acute malnutrition was 3.0% (95% CI: 2.6–3.4) and 4.2% (95% CI: 3.3–5.2) in Moramanga and Morondava, respectively. Fig 1 shows the flowchart describing the recruitment of participants.

Table 1 provides the characteristics of children included in the case-control study. Children recruited in Morondava were younger than those in Moramanga; the average age was 30.4 months (95% CI: 29.5–31.3 months) in Morondava and 33.2 months (95% CI: 32.2–34.2 months) in Moramanga. In Morondava, 82% of children had already consumed complementary food before 4 months and 44.6% had a low dietary diversity score. Child care practices were significantly better in Moramanga compared to Morondava (p<0.001): the coverage of antenatal visits in health centers during the study participants’ pregnancies was high (91% vs 64%), and the proportion of vitamin A supplementation and use of deworming medication within the 6 months prior the survey was 70.2% (vs 55.2%) and 70.6% (vs 46.2%), respectively. Additionally, 67.9% (vs 11.9%) of children had an up-to-date vaccination schedule. Almost half of the study population in both areas had respiratory symptoms (rhinitis, cough) within two weeks prior to the survey. More children had gastrointestinal symptoms in Moramanga than in Morondava, at 29.5% versus 21.2%, respectively, while fever was significantly more frequent in Morondava at 23.8% versus 18.2% in Moramanga. In Morondava, mothers were younger (56% were less than 29 years); 39% had no formal education (in Moramanga, this proportion was 11%); and more than a fourth (26%) had a low Body Mass Index (BMI) and had more than 6 pregnancies. Regarding household hygiene practices, only 11% of households in Morondava treated their drinking water and 40% had not correctly removed household waste. We did not find any significant difference in the distribution of wealth index categories between the two study sites; however, we found that more households in Morondava owned livestock.

**Table 1:** Characteristics of children included in the case-control study in Moramanga and Morondava.

Characteristics	Moramanga N = 894 N (%)	Morondava N = 932 N (%)	p-value
Child			
Gender			
Male	423 (47.3)	475 (50.9)	NS
Age group (months)			
6–11 mo	69 (7.7)	107 (11.5)	*
12–23 mo	226 (25.3)	235 (25.2)	
24–35 mo	192 (21.5)	229 (24.5)	
≥36 mo	407 (45.5)	361 (38.8)	
Breastfeed			
Yes	290 (32.4)	259 (27.8)	*
Dietary diversity score			
Low	300 (33.5)	416 (44.6)	**
Not low	594 (66.6)	516 (55.4)	
Age at introduction of complementary feeding			
<1 mo	431(48.2)	406 (43.6)	**
1–3 mo	161 (18.0)	371 (39.9)	
≥4 mo	302 (33.8)	155 (16.5)	
Birth order			
1^st^ born	214 (24.0)	197 (21.1)	*
2nd-4th	388 (43.4)	341 (36.6)	
≥5th	223 (25.0)	264 (28.3)	
Missing data	69 (7.6)	130 (14.0)	
Antenatal visit			
No	21 (2.3)	138 (14.8)	**
Traditional midwives	55 (6.1)	196 (21.0)	
Health personnel	818 (91.6)	598 (64.2)	
Birth spacing			
<24 mo	103 (11.5)	120 (12.8)	**
24–48 mo	222 (24.8)	230 (24.7)	
>48 mo	267 (29.8)	180 (19.3)	
Not applicable (1^st^ born)	214 (23.9)	197 (21.1)	
Missing data	88 (10.0)	205 (22.1)	
Low birthweight			
Yes	411 (45.9)	679 (72.8)	**
Missing data	30 (3.5)	-	
Place of delivery			
At home	511 (57.1)	734 (78.7)	**
Health center	383 (42.9)	198 (21.3)	
Deworming medication within 6 months			
Yes	632 (70.6)	431 (46.2)	**
No	184 (20.6)	376 (40.3)	
Missing data	9 (1.0)	18 (1.9)	
Not applicable (<12 mo)	69 (7.8)	107 (11.6)	
Intake of vitamin A capsule within 6 months			
Yes	628 (70.2)	515 (55.2)	**
No	255 (28.5)	399 (42.8)	
Missing data	11 (1.3)	18 (2.0)	
Immunization status^a^			
Correct	607 (67.9)	110 (11.8)	**
Not correct	257 (32.1)	818 (87.8)	
Missing data	-	4 (0.4)	
Gastrointestinal symptoms 2 weeks prior to the survey			
Yes	264 (29.5)	202 (21.7)	**
No	600 (70.5)	726 (77.9)	
Missing data	-	4 (0.4)	
Respiratory symptom 2 weeks prior to the survey			
Yes	437 (48.9)	438 (47.0)	NS
No	427 (51.1)	490 (52.6)	
Missing data		4 (0.4)	
Fever within 2 weeks prior to the survey			
Yes	163 (18.2)	221 (23.7)	*
No	701 (78.4)	707 (75.9)	
Missing data	30 (3.4)	4 (0.4)	
Maternal characteristics			
Education			
No formal education	98 (10.9)	363 (38.9)	**
Primary	535 (59.8)	435 (46.7)	
Secondary and above	261 (29.3)	133 (14.4)	
Marital status			
Married	763 (85.3)	768 (82.4)	NS
Have been married (currently separated, widowed)	102 (11.4)	135 (14.5)	
Never married	29 (3.3)	29 (3.1)	
Activity outside the household			
Yes	790 (88.3)	717 (76.9)	**
Body Mass Index status			
Thin	94 (10.5)	248 (26.6)	**
Normal	688 (77.0)	580 (62.2)	
Overweight	104 (11.6)	100 (10.7)	
Missing data	8 (0.9)	4 (0.5)	
Age (years)			
<23	159 (17.8)	304 (32.6)	**
23–28	263 (29.4)	184 (19.7)	
29–35	233 (26.1)	190 (20.4)	
≥36	239 (26.7)	181 (19.5)	
Missing data	-	73 (7.8)	
Number of pregnancies			
≤ 3	468 (52.3)	304 (32.6)	*
4–5	190 (21.2)	184 (19.7)	
≥6	166 (18.6)	190 (20.4)	
Missing data	70 (7.9)	130 (27.3)	
Number of dependent children			
≤3	524 (58.6)	443 (47.5)	**
≥4	301 (33.7)	360 (38.6)	
Missing data	69 (7.7)	129 (13.9)	
Currently pregnant			
Yes	66 (7.3)	77 (8.3)	NS
No	759 (84.9)	725 (77.8)	
Not applicable (mother not present)	69 (7.8)	130 (13.9)	
Household			
Garbage disposal^b^			
Correct	752 (84.1)	562 (60.3)	**
Not correct	142 (15.9)	370 (39.7)	
Storage of drinking water			
Outside	32 (3.5)	26 (2.8)	NS
Inside	845 (94.5)	902 (96.8)	
Missing data	17 (2.0)	4 (0.4)	
Protection of drinking water			
Yes	559 (62.5)	461 (49.5)	**
No	321 (36.0)	467 (50.1)	
Missing data	14 (1.5)	4 (0.4)	
Treatment of drinking water			
Yes	520 (58.1)	105 (11.3)	**
Wealth index			
Poorest	237 (26.5)	259 (27.8)	NS
Poorer	211 (23.5)	230 (24.7)	
Wealthier	223 (25.0)	220 (23.6)	
Wealthiest	223 (25.0)	223 (23.9)	
Ownership of livestock			
Yes	716 (80.0)	805 (86.4)	**
Ownership of arable land			
Yes	865 (96.7)	876 (94.0)	*
Monthly budget for food^c^			
Lowest	187 (21.0)	241 (23.0)	NS
Lower	245 (27.4)	235 (25.2)	
Higher	125 (14.0)	118 (12.7)	
Highest	332 (37.1)	338 (39.1)	
Missing data	5 (0.5)		
Food consumption score level			
Poor	58 (6.5)	51 (5.5)	**
Limit	489 (54.7)	259 (27.8)	
Acceptable	347 (38.9)	622 (66.7)	
Total	894	932	

*: significant;

**: p<10^−3^; statistical tests were conducted with complete data;

^a^: Immunization status was adequate if the child had completed all recommended vaccinations according to his/her age;

^b^: Household garbage disposal was correct if the household used dustbins or burned the garbage;

^c^: We divided the monthly budget for food by 4 categories (quartile), “lowest”: <2,500 Ariary (local money), “lower”: 2,500–3,599 Ariary, “higher”: 3,600–4,800 Ariary and “highest”: >4,800 Ariary.

The results of stool examinations for intestinal parasites showed the presence of at least one investigated parasite in 23.6% of the enrolled children [95% CI: 21.3–26.0]. The prevalence of parasitic infections was 28.4% [95% CI: 25.5–31.4] in Moramanga and 18.3% [95% CI: 16.0–21.0] in Morondava. The most common identified parasite in Moramanga was *Ascaris lumbricoides* (16.1%), followed by *Trichuris Trichiura (3*.*8%)*. In Morondava, *Entamoeba coli* (6%) and *Hymenolepis nana (4%)* were the most prevalent. Infections with more than one parasite (mixed infection) were found in 11% of infected children (2.5% of all studied children), 13.4% in Moramanga and 7.6% in Morondava. Table 2 provides information on the parasitic carriage in children in the study at the two study sites.

**Table 2:** Parasitic carriage of children included in the case-control study in Moramanga and Morondava.

Intestinal parasites	Moramanga (n = 894) N (%)	Morondava (n = 932) N (%)	p-value
*Ascaris lumbricoides*	145 (16.2)	11 (1.2)	**
*Trichuris trichiura*	34 (3.8)	2 (0.2)	**
*Giardia intestinalis*	14 (1.6)	12 (1.3)	NS
*Hymenolepis nana*	2 (0.2)	38 (4.1)	**
Other amoebae	47 (5.2)	100 (10.7)	*
Other helminths	3 (0.3)	3 (0.3)	NS
Cryptosporidies	15 (1.7)	9 (0.9)	NS
*Cyclospora* spp.	10 (1.1)	1 (0.2)	*
*Isospora* spp.	-	1 (0.2)	NS

*: significant;

**: p<10^−3^; statistical tests were conducted with complete data; NS: not significant

### Determinants of stunting

In Moramanga, household wealth index, age group and infection with *Trichuris trichiura* were determinants of stunting that remained in the final adjusted model (Table 3). Compared to children from the wealthiest households, those from the poorest households were more likely to be stunted. Household wealth index within the 1^st^, 2^nd^ and 3^rd^ quartile compared with the 4^th^ quartile was associated with 2.3 (95% CI: 1.6–3.4), 2.3 (95% CI: 1.5–3.4) and 1.2 (95% CI: 0.8–1.7) odds of stunting, respectively. The odds of being stunted increased 2.4 times (95% CI: 1.1–5.3) for children who had *Trichuris trichiura* infection compared to children without infection. Child’s age was also found to be an independent predictor of stunting, with older children more likely to be stunted than those below 12 months: adjusted OR of 4.0 (95% CI: 2.2–7.5), 3.6 (95% CI: 1.9–6.7) and 2.7 (95% CI: 1.5–4.8) in children aged 12–23, 24–35 and ≥36 months, respectively.

**Table 3:** Final model estimates for determinants of stunting in children 6 to 59.9 months, HDSS of Moramanga (n = 894).

Characteristics	Stunting		Unadjusted OR (95% CI)	*p*-value	Adjusted OR (95% CI)	p-value
	Yes, N (%)	No, N (%)				
Child characteristics						
Age group (months)						
6–11 mo	17 (24.7)	52 (75.3)	1	-	1	-
12–23 mo	126 (55.7)	100 (44.2)	3.8 (2.1–7.1)	0.000	4.0 (2.2–7.5)	0.000
24–35 mo	101 (52.6)	91 (47.4)	3.4 (1.8–6.3)	0.000	3.6 (1.9–6.7)	0.000
≥36 mo	187 (46.0)	220 (54.0)	2.6 (1.4–4.6)	0.001	2.7 (1.5–4.8)	0.001
Infection by *Trichuris trichiura*						
No	407 (43.3)	453 (52.7)	1	-	1	-
Yes	24 (70.6)	10 (29.4)	2.7 (1.3–5.6)	0.01	2.4 (1.1–5.3)	0.002
Household characteristic						
Wealth index						
Wealthiest	82 (36.8)	141 (63.2)	1	-	1	-
Wealthier	92 (41.3)	131 (58.7)	1.2 (0.8–1.8)	0.4	1.2 (0.8–1.7)	0.5
Poorer	122 (57.8)	89 (42.2)	2.3 (1.6–3.5)	0.000	2.3 (1.5–3.4)	0.000
Poorest	135 (57.0)	102 (43.0)	2.3 (1.6–3.3)	0.000	2.3 (1.6–3.4)	0.000

OR: odds ratio; 95% CI: 95% confidence interval

In the Morondava study site, child’s age, mother’s working status, child’s birth weight and birth spacing were determinants of stunting (Table 4). The odds of stunting in children aged 12–23 months and 24–35 months relative to younger children (6–11 mo) were 2.5 times (95% CI: 1.5–4.0) and 1.8 times greater (95% CI: 1.1–2.9). Birth weight remained an underlying determinant of stunting; children born with low birthweight were 1.6 times (95% CI: 1.1–2.1) more likely to be stunted. Other underlying predictors related to mothers were birth spacing and mother’s working status. Children born after a longer birth interval were less likely to be stunted; specifically, those with birth intervals of 24–48 mo and ≥48 mo were 50% (aOR: 0.5; 95% CI: 0.3–0.8) and 60% (aOR: 0.4; 95% CI: 0.3–0.7) less likely to be stunted, respectively, compared with children with a birth interval less than 24 months. Children of working mothers were 1.7 times (aOR: 1.7; 95% CI: 1.2–2.5) more likely to be stunted. Having respiratory symptoms within 2 weeks before the survey was a protective factor for stunting (aOR: 0.7; 95% CI: 0.5–0.9).

**Table 4:** Final model estimates for determinants of stunting in children 6 to 59.9 months, municipalities of Bemanonga-Morondava (n = 932).

Characteristics	Stunting		Unadjusted OR (95% CI)	*p*-value	Adjusted OR (95% CI)	*p*-value
	Yes, N (%)	No, N (%)				
Child characteristics						
Age group (months)						
6–11 mo	35 (32.7)	72 (67.3)	1	-	1	-
12–23 mo	133 (56.6)	102 (43.4)	2.7 (1.7–4.3)	0.0000	2.5 (1.5–4.0)	0.0002
24–35 mo	115 (50.2)	114(49.8)	2.1 (1.3–3.3)	0.002	1.8 (1.1–2.9)	0.02
≥36 mo	137 (37.9)	224 (62.1)	1.2 (0.8–1.9)	0.3	1.0 (0.6–1.7)	0.8
Low birthweight						
No	92 (36.4)	161 (63.6)	1	-	1	-
Yes	327 (68.8)	148 (31.2)	1.6 (1.2–2.2)	0.001	1.6 (1.1–2.1)	0.004
Missing	1 (25.0)	3 (75.0)	0.6 (0.0–5.6)	0.6	0.4 (0.0–4.0)	0.4
Birth spacing						
<24 mo	69 (57.5)	51 (42.5)	1	-	1	-
24–48 mo	96 (41.7)	134 (58.3)	0.5 (0.3–0.8)	0.005	0.5 (0.3–0.8)	0.003
≥48 mo	71 (39.4)	109 (60.6)	0.5 (0.3–0.7)	0.002	0.4 (0.3–0.7)	0.001
First born	88 (44.7)	109 (55.3)	0.6 (0.4–0.9)	0.02	0.6 (0.4–0.9)	0.04
Missing	96 (46.8)	109 (53.2)	0.6 (0.4–1.0)	0.06	0.7 (0.4–1.1)	0.2
Mother’s characteristics						
Mother’s working status						
Not working	81 (37.7)	134 (62.3)	1	-	1	-
Working	339 (47.3)	378 (52.7)	1.5 (1.1–2.0)	0.01	1.7 (1.2–2.5)	0.001
Occurrence of respiratory symptoms within the previous 2 weeks						
No	241 (49.2)	249 (50.8)	1	-	1	-
Yes	178 (40.6)	260 (59.4)	0.7 (0.5–0.9)	0.009	0.7 (0.5–0.9)	0.005
Missing	1 (25.0)	3 (75.0)	0.3 (0.0–3.3)	0.3	NA	NA

OR: odds ratio; 95% CI: 95% confidence interval; NA: not available

## Discussion

In this study, which was conducted in rural areas of Moramanga and Morondava, we examined the determinants of stunting in children aged 6 to 59.9 months. This study confirmed that stunting was highly prevalent in these two areas of Madagascar. In the HDSS of Moramanga, we found evidence that child’s age, *Trichuris trichiura* infection and household wealth index were predictors of child stunting. Data from the municipalities of Bemanonga Morondava, suggested that increases in child’s age, mother’s working status, and low birth weight were risk factors for stunting, whereas increased birth spacing was a protective factor.

Although the prevalence of stunting was high in both study areas, it was higher in Moramanga, with more than 1 in 2 children being affected (52.8%; 95% CI: 51.7–54.0). Our data also suggested that parasite intestinal carriage was more frequent in children enrolled in Moramanga than those in Morondava, whereas children’s feeding, household hygiene and health care practices seemed to be poorer in Morondava.

Similar associations between child’s age and stunting have been found in other studies in Madagascar [16], Libya [25], Nigeria [26] and Kenya [27]. Stunting was also associated with age range (12 mo to 35 mo) in both study locations; this finding was similar to those of another study, in which the strongest association between age and stunting was found in children between 12 and 23 mo of age [28]. The factors increasing the risk of stunting in this period may include weaning, which coincides with low-quality food supplementation, loss of passive immunity and exposure to unsanitary conditions, which increases the risk of infections [29–31]. In Moramanga, the association between stunting and age was stronger and seemed to continue after 36mo, this could be attributed to the parasitic infection which remains present after 36 months.

Our statistical analyses in the DHSS of Moramanga suggested that the likelihood of stunting was higher in children infected with *Trichuris trichiura*; this finding is supported by other studies, although most of them were performed among school-aged children [32–35]. *Trichuris trichiura* can remain in the digestive tract for more than 3 years, and this chronicity of infection could ultimately affect the child’s growth [36]. Inflammations caused by parasitic infections and environmental enteric dysfunction, which are commonly widespread in the context of poor environments similar to those in our study area, may exacerbate growth failure and contribute to stunting [37]. The findings in Moramanga revealed that children from less wealthy households were at greater risk of stunting than those from wealthy households. This significant association between wealth index and stunting has been cited in previous studies [26,27,38]. Household socioeconomic status determines the availability of good and nutritious foods for children’s growth [26], access to health services and likely children’s education level. The findings from the HDSS of Moramanga suggested that there is a clear need to strengthen interventions to reduce economic inequalities and poverty to improve children’s nutritional status. In addition, interventions that could promote individual hygiene practices, community sanitation coverage and mother’s compliance with and access to public health services could have an impact on child’s growth. Infection with soil-transmitted helminthes can be disrupted by good hygiene practices [39] and good sanitation coverage by preventing environmental contamination; use of deworming programs by mother and children could have a greater impact on parasite transmission community-wide. Based on data from other regions, there is evidence that sanitation coverage has a strong impact on child height [40,41], and a recent publication from developing countries including Madagascar revealed that unimproved sanitation was the leading risk factor for stunting, with 12.3% (10.1–14.9) of the prevalence of stunting being attributed to this factor in Madagascar [42].

In the municipalities of Bemanonga in Morondava, the main determinants of stunting were those related to mother’s characteristics. Our data were consistent with studies that have shown that children’s nutritional status is related to maternal presence and mother’s reproductive status [39,43,44]. Children whose mothers worked outside of the home were likely to be stunted (aOR: 1.7, CI 95%:1.2–2.5). Maternal time allocation may affect a child’s nutritional status by determining the time spent caring for the child. Our findings in Morondava confirmed the importance of activities that contribute to reducing the workload on women, as we found that maternal absence affected child’s nutritional status. Activities outside the household may provide an opportunity for women to increase their income in some situations; however, reasonable workloads and time availability determine mothers’ or caregivers’ ability to provide adequate childcare [45]. Sharing responsibilities for childcare with men might be an alternative; however, a better understanding of the local cultural norms defining women’s and men’s reproductive roles is necessary for programs to be successful [43]. We also found that birthweight and preceding birth interval were predictors of stunting. Children whose mothers perceived them to be small at birth showed a relatively higher risk of being stunted. Stunting begins in utero and continues for at least the first 2 years of postnatal life [46], and it has been estimated that 20% of stunting has origins in utero [47]. Previous studies have also shown that small newborns do not seem to demonstrate marked catch-up in growth during infancy [16,27,48–50]. The link between low birth weight and growth failure could be explained by an increased vulnerability to infection, which can result in poor physical growth [51,52]. The main reason for low birth weight in developing countries is intra-uterine growth retardation (IUGR) [53]. In developing countries, IUGR is attributable to mother’s malnutrition, low body mass index and low weight gain during pregnancy [54,55], anemia and iron deficiency [56], and early pregnancy during adolescence, when mothers are still growing [57,58]. Furthermore, our data corroborate earlier evidence that closely spaced births are associated with stunting [39,43,44]; we found that adequate birth spacing (≥24 months) appeared beneficial for child height. This finding may be explained by a greater risk of adverse birth outcomes including low birth weight in second children [59–61]; maternal depletion has been proposed as a possible etiology for the association between short birth spacing after a live birth and low birth weight [62]. Improved promotion of child spacing and family planning could reduce maternal burden and, indirectly, stunting; in Madagascar, the proportion of married women using modern contraceptives was found to be 33% [11]. In the Menabe region, approximately 15% of children with available data on birth weight weighed less than 2500 g and 21.2% of children were perceived to be small by their mother [11]. Surprisingly, we found that children who had experienced respiratory symptoms within the 2 weeks prior to the survey were at a lower risk of stunting. Numerous studies have shown that recurrent infections such as diarrhea and respiratory illnesses can impair growth [63,64]. This result might be due to unmeasured factors that were linked to the characteristics of children who had respiratory symptoms. In addition, occurrence of respiratory symptoms in the 2 past weeks may not be a good indicator of stunting, which is a long process. In contrast to national results regarding the determinants of stunting [16], we did not find an association between stunting and gender except in children under 24 mo in the DHSS of Moramanga, which suggested that 60% of girls under 24 mo were not stunted.

This study has some limitations. First, the case-control design did not enable investigations of causation only associations. However, most of the observed determinants have been proven by other study designs with strong plausibility and consistency. Second, data on birth size was based on mother’s perceptions; however, this metric is considered a suitable proxy for birthweight [65]. During the last survey conducted in Madagascar, only 14.5% of children had available data on birth weight [11]. Third, the amount of missing data for the birth spacing variable in Morondava was relatively high (22%); thus, we included observations with missing values as an additional category in our analysis. We found the same determinants when the analysis was conducted by excluding missing data, with the exception of the occurrence of respiratory symptoms in the past 2 weeks, which appeared to be statistically significant in the model with missing values. Fourth, the technique used to identify opportunistic parasites lacked sensitivity, and thus we may have underestimated their prevalence. Finally, we do not have data on the non-responders, however the collection of high quality data was ensured through rigorous training as well as the development of data collection and data entry procedures.

In conclusion, our analysis confirms that stunting remains a major public health problem in the HDSS of Moramanga and in the municipalities of Bemanonga, Morondava. After many years of neglect, stunting is now considered as a major global health priority. In Madagascar, the new nutrition plan (Plan National d’Action III 2017-2021-PNAN III) aims at reducing the prevalence of chronic malnutrition from 47% to 38% by 2021. The three main strategies to reach this objective are nutrition-specific and nutrition-sensitive interventions combined with better governance. All the stunting determinants identified in this study are targeted by the current PNAN III and are key components of the Sustainable Development Goal e.g to eradicate poverty (Goal 1), to ensure good health and well-being (Goal 3), to empower women and girls (Goal 5) and to ensure availability of clean water and sanitation (Goal 6). Programs with solid health access, safety net, hygiene and sanitation components should efficiently reduce stunting in Madagascar. However a strong political commitment matched with multi-sectoral collaborations and wider program coverage are necessary. This broad commitment is more than ever needed to cope with the new socio-economic challenges adding to the double burden of malnutrition (both under and overnutrition) due to the increased urbanization and shifts in diet and lifestyle that emerged in developing countries.

## Supporting information

S1 Table De-identified data. (XLSX)

S1 Text Missing data. (DOCX)

S2 Text First questionnaire (in English). (DOCX)

S3 Text Second questionnaire (in English). (DOCX)

S4 Text Second questionnaire in the original language. (DOCX)

S5 Text First questionnaire in the original language. (DOCX)
