# Correction: The importance of public health, poverty reduction programs and women's empowerment in the reduction of child stunting in rural areas of Moramanga and Morondava, Madagascar

**DOI:** 10.1371/journal.pone.0189747

**Published:** 2017-12-11

**Authors:** Chitale Rabaoarisoa Remonja, Rado Rakotoarison, Nivo Heritiana Rakotonirainy, Reziky Tiandraza Mangahasimbola, Alain Berthin Randrianarisoa, Ronan Jambou, Inès Vigan-Womas, Patrice Piola, Rindra Vatosoa Randremanana

The first author's name is incorrect. The correct name is: Chitale Rabaoarisoa Remonja. The correct citation is: Remonja CR, Rakotoarison R, Rakotonirainy NH, Mangahasimbola RT, Randrianarisoa AB, Jambou R, et al. (2017) The importance of public health, poverty reduction programs and women’s empowerment in the reduction of child stunting in rural areas of Moramanga and Morondava, Madagascar. PLoS ONE 12(10): e0186493. https://doi.org/10.1371/journal.pone.0186493

## References

[1] Rabaoarisoa CR, Rakotoarison R, Rakotonirainy NH, Mangahasimbola RT, Randrianarisoa AB, Jambou R, et al (2017) The importance of public health, poverty reduction programs and women’s empowerment in the reduction of child stunting in rural areas of Moramanga and Morondava, Madagascar. PLoS ONE 12(10): e0186493 https://doi.org/10.1371/journal.pone.0186493 29045444 10.1371/journal.pone.0186493 PMC5646813

