# Supplementary material for: The importance of public health, poverty reduction programs and women’s empowerment in the reduction of child stunting in rural areas of Moramanga and Morondava, Madagascar
Source: PLoS One. 2017 Oct 18;12(10):e0186493. doi: 10.1371/journal.pone.0186493 (PMC5646813; doi:10.1371/journal.pone.0186493)
Supplement: S1 Text — (DOCX) [file pone.0186493.s002.docx]

**Missing data**

In Moramanga, <10% of the sample had missing data for variables entered into the first backward stepwise model. Birth interval (9.8%, n=88) and number of children cared for by the mother (7.7%, n=69) had the most missing values. The remaining variables with missing data accounted for less than 4% of the dataset (mother’s BMI: 0.8%, n=8; child’s birth weight: 3.3%, n=30).

In Morondava, birth spacing had the most missing data (22%, n=205). The remaining variables with missing data included in the multivariate analysis accounted for less than 1% of the dataset (occurrence of gastrointestinal symptoms, respiratory symptoms and fever; n=4).
