# Supplementary material for: The importance of public health, poverty reduction programs and women’s empowerment in the reduction of child stunting in rural areas of Moramanga and Morondava, Madagascar
Source: PLoS One. 2017 Oct 18;12(10):e0186493. doi: 10.1371/journal.pone.0186493 (PMC5646813; doi:10.1371/journal.pone.0186493)
Supplement: S2 Text — (DOCX) [file pone.0186493.s003.docx]

**Questionnaire on the feeding practices and the care of the children**

| *Child identification* | | | | | | | | | | | | | |
| --- | --- | --- | --- | --- | --- | --- | --- | --- | --- | --- | --- | --- | --- |
| **P1a** | Name and first name of the child : **Nomenf** | | | | | | | | | | | | |
| **P1b** | Identification number of the child: \|__\|__\|__\|__\|__\|__\|__\|__\|__\|__\|__\|__\|__\| **Idenf** | | | | | | | | | | | | |
| **P2** | Number of sample **Nprel** | | | | | | | | | | | | |
| **P3** | Child’s sex :(1) Male ; (2) Female | | | | | | | | | \|__\| **Senf** | | | |
| **P4a**  **P4b** | Date of the birth of the child :  (1) Verified (2) Declared (3) Not known (If P4b=1 or 2 →P5) | | | | | | | | | \|__\|__\|/\|__\|__\|/\|__\|__\| **Datenf**  \|__\| **Datenfvd** | | | |
| **P4c** | Age in month | | | | | | | | | \|__\|__\| **Agenf** | | | |
| **P4d** | Is the child a twin? (1) Yes (0) No | | | | | | | | | \|__\| **Jumeau** | | | |
| **P5** | Group of the child in the screening (1) MC (2) MA (3) NM | | | | | | | | | \|__\| **Grenfdep** | | | |
| *Anthropometrics measurements* | | | | | | | | | | | | | |
| **Mother or person who usually takes care of the child** | | | | | | | | | | | | | |
| **P6a**  **P6b**  **P6c**  **P6** | *Weight of the mother :*  - Measure 1  - Measure 2  - Measure 3  →Average | | | | | | | | | \|__\|__\|,\|__\|__\| kg **Poidmer 1**  \|__\|__\|,\|__\|__\| kg **Poidmer 2**  \|__\|__\|,\|__\|__\| kg **Poidmer 3**  \|__\|__\|,\|__\|__\| kg **Poidmer** | | | |
| **P7a**  **P7b**  **P7c**  **P7** | *Height of the mother:*  - Measure 1  - Measure2  - Measure 3  →Average | | | | | | | | | \|__\|__\|__\|,\|__\| cm **Tailmer 1**  \|__\|__\|__\|,\|__\| cm **Tailmer 2**  \|__\|__\|__\|,\|__\| cm **Tailmer 3**  \|__\|__\|__\|,\|__\| cm **Tailmer** | | | |
| **P7d** | Does the mother present a disability which can affect the anthropometrics measurements? (1) Yes (0) No | | | | | | | | | \|__\| **Handphymer** | | | |
| **Child** | | | | | | | | | | | | | |
| **P8a**  **P8b**  **P8c**  **P8** | *Weight of the child :*  - Measure 1  - Measure 2  - Measure 3  →Average | | | | | | | | | \|__\|__\|,\|__\|__\| kg **Poidenf 1**  \|__\|__\|,\|__\|__\| kg **Poidenf 2**  \|__\|__\|,\|__\|__\| kg **Poidenf 3**  \|__\|__\|,\|__\|__\| kg **Poidenf** | | | |
| **P9a**  **P9b**  **P9c**  **P9** | *Height of the child :*  - Measure 1  - Measure2  - Measure 3  →Average | | | | | | | | | \|__\|__\|__\|,\|__\| cm **Taillenf 1**  \|__\|__\|__\|,\|__\| cm **Taillenf 2**  \|__\|__\|__\|,\|__\| cm **Taillenf 3**  \|__\|__\|__\|,\|__\| cm **Taillenf** | | | |
| **P10a**  **P10b**  **P10c**  **P10** | *Mid-Upper Arm Circumference:*  - Mesure 1  - Mesure2  - Mesure 3  →Mean | | | | | | | | | \|__\|__\|,\|__\| cm **Pbenf 1**  \|__\|__\|,\|__\| cm **Pbenf 1**  \|__\|__\|,\|__\| cm **Pbenf 3**  \|__\|__\|,\|__\| cm **Pbenf** | | | |
| **P11a** | Nutritional status of the child in the time of the survey :  (1) MC (stunted) (2) MA (wasted) (3) NM (non-malnourished) | | | | | | | | | \|__\| **Grenfenq** | | | |
| **P11b** | Does the child present a disability which can affect the anthropometrics measurements? (1) Yes (0) No | | | | | | | | | \|__\| **Handphyenf** | | | |
| *Diet of the child* | | | | | | | | | | | | | |
| **P12**  **P12a**  **P12b** | Since the screening, did you change the diet of this child?  (1) Yes(0) No  - If yes, why? (1) My child grew up in age (2) By following the advice which you had given  (3) Others (to be specified)  - If yes, what did you change?  (1) Increase the quantity/the number of the meal (1) Yes (0) No  (2) Diversify the food (1) Yes (0) No  (3) Introduce (add) new food (1) Oui (0) Non  - Food 1  - Food 2  - Food 3  (4) Others (to be specified) | | | | | | | | | \|__\| **Charegenf**  \|__\| **Rchareg**  ……………….. **Rchareg_aut**  \|__\| **Modcha**  \|__\|  \|__\|  ………………..**Alim 1**  ………………..**Alim 2**  ………………..**Alim 3**  ………………..**Modchaut** | | | |
| **P13a**  **P13b** | - How long after birth was the child put in the breast for the first time? (1) Less than one hour  (2) Less than 24 hours (3) 24 hours and more (4) He was never breast-fed (5) Doesn’t remember  - If 3 or 4, why didn’t you breast-feed him during the first day? (1) Delay of milky rise (2) You think that the first milk is not good for the baby?  (3) You think baby’s body has to cleaned itself at first (4) Other reasons (to be specified) | | | | | | | | | \|__\| **Seinpref**  \|__\|__\| **Rnseinpref**  …………… **Arnseinpref** | | | |
| **P13c**  **P13d** | - After his/her birth, did you give anything else to the child before breast-feeding? (1) Yes (0) No  - If yes, what did you give? (1) some infant formula  (2) some water (3) some sugar water (4) some tea/coffee  (5) some ranom-bary/some ranon’ampango (6) Something else (to be specified) | | | | | | | | | \|__\| **Consavlaim**  \|__\|__\| **Alcons**  …………….**Autalcons** | | | |
| **P14**  **P14a**  **P14b**  **P14c** | Yesterday, did the child take the breast? (1) Yes ; (0) No  - If yes, how many times : (1) less than 5 times ; (2) from 5 to 9 times; (3) from 10 to 14 times; (4) 15 times and more; (5) nsp  - If not, why? (1) already weaned ; (2) sick child ;  (3) Mother separated from the child/ busy ; (4) Sick mother ; (5) Dead mother ; (6) Other (to be specified)  - If the child is already weaned (*Rnsein=1*), How old (in month) was him/her when you weaned him/her? | | | | | | | | | \|__\| **Prisein**  \|__\| **Freprisein**  \|__\| **Rnsein**  **Rnseinaut**  \|__\|__\| **Agesevra** | | | |
| **P15** | For those who breast-feed, at which moment do you breast-feed the child? (1) at he request(2) at the fixed moments ? | | | | | | | | | \|__\| **Momall** | | | |
| **P16** | Yesterday, did the child receive food by means of a feeding bottle? (1) Yes ; (0) No | | | | | | | | | \|__\| **Biberon** | | | |
| Yesterday, what type of dish ate your child in the various meals? | | | | | | | | | | | | | |
|  | | Porridge**P17a** | | | Snack **P17b** | Fruit **P17c** | | | Special meal **P17d** | | | Familial meal **P17e** | |
| *Breakfast*  **Pdejenf** | | (1) Yes (0) No | | | (1) Yes (0) No | (1) Yes (0) No | | | (1) Yes (0) No | | | (1) Yes (0) No | |
| *Snack/morning*  **Gmatenf** | | (1) Yes (0) No | | | (1) Yes (0) No | (1) Yes (0) No | | | (1) Yes (0) No | | | (1) Yes (0) No | |
| *Lunch*  **Dejenf** | | (1) Yes (0) No | | | (1) Yes (0) No | (1) Yes (0) No | | | (1) Yes (0) No | | | (1) Yes (0) No | |
| *Snack/afternoon* **Gapremenf** | | (1) Yes (0) No | | | (1) Yes (0) No | (1) Yes (0) No | | | (1) Yes (0) No | | | (1) Yes (0) No | |
| *Dinner* **Dinenf** | | (1) Yes (0) No | | | (1) Yes (0) No | (1) Yes (0) No | | | (1) Yes (0) No | | | (1) Yes (0) No | |
|  | | | | | | | | | | | | | |
|  | | | | | | | | | | | | | |
| Food | | At what age in month did he begin to consume of? | | | Yesterday, how many times did the child consume? (Note 0 if the child did not consume) | | What quantity did the child consume yesterday? (in spoons, bowls, glass, jar, number, piece) | | | | | How many days during last week? (Note 0 if the child did not consume) | |
| **Céréales** | | | | | | | | | | | | | |
| Ranon’ampango | | **P18a** \|__\|__\|,\|__\| **Rpagenf** | | | **P18b** \|__\| **Rpvenf** | | **Quarpvenf**  \|__\|__\|,\|__\| glass | | | | | **P18c** \|__\| **Rpdsenf** | |
| Porridge | | **P19a** \|__\|__\|,\|__\| **Bouagenf** | | | **P19b** \|__\| **Bouvenf** | | **Quabouvenf**  \|__\|__\|,\|__\| bowl | | | | | **P19c** \|__\| **Boudsenf** | |
| Boiled rice | | **P20a** \|__\|__\|,\|__\| **Vsagenf** | | | **P20b** \|__\| **Vsvenf** | | **Quavsvenf**  \|__\|__\|,\|__\| bowl | | | | | **P20c** \|__\| **Vsdsenf** | |
| Dry rice | | **P21a** \|__\|__\|,\|__\| **Vmagenf** | | | **P21b** \|__\| **Vmvenf** | | **Quavmvenf**  \|__\|__\|,\|__\| bowl | | | | | **P21c** \|__\| **Vmdsenf** | |
| Corn | | **P22a** \|__\|__\|,\|__\| **Maiagenf** | | | **P22b** \|__\| **Maivenf** | | **Quamaivenf**  \|__\|__\|,\|__\| spoon | | | | | **P22c** \|__\| **Maidsenf** | |
| Bread | | **P23a** \|__\|__\|,\|__\| **Painagenf** | | | **P23b** \|__\| **Painvenf** | | **Quapainvenf**  \|__\|__\|,\|__\| piece | | | | | **P23c** \|__\| **Paindsenf** | |
| Biscuit | | **P24a** \|__\|__\|,\|__\| **Bisagenf** | | | **P24b** \|__\| **Bisvenf** | | **Quabisvenf**  \|__\|__\|,\|__\| piece | | | | | **P24c** \|__\| **Bisdsenf** | |
| Dough | | **P25a** \|__\|__\|,\|__\| **Patagenf** | | | **P25b** \|__\| **Patvenf** | | **Quapatvenf**  \|__\|__\|,\|__\| bowl | | | | | **P25c** \|__\| **Patdsenf** | |
| Cooking water of cereal | | **P26a** \|__\|__\|,\|__\| **Bceragenf** | | | **P26b** \|__\| **Bcervenf** | | **Quabcervenf**  \|__\|__\|,\|__\| glass | | | | | **P26c** \|__\| **Bcerdsenf** | |
| Others cereals  **Atcerenf** | | **P27a** \|__\|__\|,\|__\|  **Atceragenf** | | | **P27b** \|__\| **Atcervenf** | | **Quatcervenf**  \|__\|__\|,\|__\| | | | | | **P27c** \|__\| **Atcerdsenf** | |
| **Roots and tubers** | | | | | | | | | | | | | |
| Cassava | | **P28a** \|__\|__\|,\|__\| **Managenf** | | | **P28b** \|__\| **Manvenf** | | **Quamanvenf**  \|__\|__\|,\|__\| piece | | | | | **P28c** \|__\| **Mandsenf** | |
| Yam/Sweet potato | | **P29a** \|__\|__\|,\|__\| **Pagenf** | | | **P29b** \|__\| **Pavenf** | | **Quapavenf**  \|__\|__\|,\|__\| piece | | | | | **P29c** \|__\| **Padsenf** | |
| Taro | | **P30a** \|__\|__\|,\|__\| **Tagenf** | | | **P30b** \|__\| **Tavenf** | | **Quatavenf**  \|__\|__\|,\|__\| piece | | | | | **P30c** \|__\| **Tadsenf** | |
| Potato | | **P31a** \|__\|__\|,\|__\| **Potagenf** | | | **P31b** \|__\| **Potvenf** | | **Quapotvenf**  \|__\|__\|,\|__\| piece | | | | | **P31c** \|__\| **Potdsenf** | |
| Cooking water of roots/  tubers | | **P32a** \|__\|__\|,\|__\| **Brtagenf** | | | **P32b** \|__\| **Brtvenf** | | **Quabrtvenf**  \|__\|__\|,\|__\| glass | | | | | **P32c** \|__\| **Brtdsenf** | |
| Others roots/tubers **Aurtenf** | | **P33a**\|__\|__\|,\|__\|  **Aurtagenf** | | | **P33b** \|__\| **Aurtvenf** | | **Quaurtvenf**  \|__\|__\|,\|__\| piece | | | | | **P33c** \|__\| **Aurtdsenf** | |
| **Legumes** | | | | | | | | | | | | | |
| Bean | | **P34a** \|__\|__\|,\|__\| **Haragenf** | | | **P34b** \|__\| **Harvenf** | | **Quaharvenf**  \|__\|__\|,\|__\| spoon | | | | | **P34c** \|__\| **Hardsenf** | |
| Mung bean | | **P35a** \|__\|__\|,\|__\|  **Ambagenf** | | | **P35b** \|__\| **Ambvenf** | | **Quambvenf**  \|__\|__\|,\|__\| spoon | | | | | **P35c** \|__\| **Ambdsenf** | |
| Pea | | **P36a** \|__\|__\|,\|__\| **Pcagenf** | | | **P36b** \|__\| **Pcvenf** | | **Quapcvenf**  \|__\|__\|,\|__\| spoon | | | | | **P36c** \|__\| **Pcdsenf** | |
| Cowpea | | **P37a** \|__\|__\|,\|__\| **Niagenf** | | | **P37b** \|__\| **Nivenf** | | **Quanivenf**  \|__\|__\|,\|__\| spoon | | | | | **P37c** \|__\| **Nidsenf** | |
| Lentils | | **P38a** \|__\|__\|,\|__\| **Lenagenf** | | | **P38b** \|__\| **Lenvenf** | | **Qualenvenf**  \|__\|__\|,\|__\| spoon | | | | | **P38c** \|__\| **Ledsenf** | |
| Voandzou | | **P39a** \|__\|__\|,\|__\| **Voagenf** | | | **P39b** \|__\| **Vovenf** | | **Quavovenf**  \|__\|__\|,\|__\| spoon | | | | | **P39c** \|__\| **Vodsenf** | |
| Peanut | | **P40a** \|__\|__\|,\|__\| **Aragenf** | | | **P40b** \|__\| **Arvenf** | | **Quarvenf**  \|__\|__\|,\|__\| spoon | | | | | **P40c** \|__\| **Ardsenf** | |
| Cooking water of legumes | | **P41a** \|__\|__\|,\|__\| **Blegagenf** | | | **P41b** \|__\| **Blegvenf** | | **Quablegvenf**  \|__\|__\|,\|__\| glass | | | | | **P41c** \|__\| **Blegdsenf** | |
| Other legumes  **Aulegenf** | | **P42a**\|__\|__\|,\|__\| **Aulegagenf** | | | **P42b** \|__\| **Aulegvenf** | | **Quaulegvenf**  \|__\|__\|,\|__\| spoon | | | | | **P42c** \|__\| **Aulegdsenf** | |
| **Milk and dairy products** | | | | | | | | | | | | | |
| Milk other than breastmilk | | **P43a** \|__\|__\|,\|__\| **Laiagenf** | | | **P43b** \|__\| **Laivenf** | | **Qualaivenf**  \|__\|__\|,\|__\| glass | | | | | **P43c** \|__\| **Laidsenf** | |
| Yoghurt | | **P44a** \|__\|__\|,\|__\| **Yoagenf** | | | **P44b** \|__\| **Yovenf** | | **Quayovenf**  \|__\|__\|,\|__\| pot | | | | | **P44c** \|__\| **Yodsenf** | |
| Other dairy products **Aulenf** | | **P45a** \|__\|__\|,\|__\| **Aulagenf** | | | **P45b** \|__\| **Aulvenf** | | **Quaulvenf**  \|__\|__\|,\|__\| | | | | | **P45c** \|__\| **Auldsenf** | |
| **Meat, Poultry, Fish and its by-products** | | | | | | | | | | | | | |
| Beef /derivative | | **P46a** \|__\|__\|,\|__\| **Boagenf** | | | **P46b** \|__\| **Bovenf** | | **Quabovenf**  \|__\|__\|,\|__\| piece | | | | | **P46c** \|__\| **Bodsenf** | |
| Pork/derivative | | **P47a** \|__\|__\|,\|__\| **Poagenf** | | | **P47b** \|__\| **Povenf** | | **Quapovenf**  \|__\|__\|,\|__\| piece | | | | | **P47c** \|__\| **Podsenf** | |
| Sausage | | \|__\|__\|,\|__\| **Sauagenf** | | | \|__\| **Sauvenf** | | **Quasauvenf**  \|__\|__\|,\|__\| piece | | | | | \|__\| **Saudsenf** | |
| Poultry | | **P48a** \|__\|__\|,\|__\| **Volagenf** | | | **P48b** \|__\| **Volvenf** | | **Quavolvenf**  \|__\|__\|,\|__\| piece | | | | | **P48c** \|__\| **Voldsenf** | |
| Giblets | | **P49a** \|__\|__\|,\|__\| **Abagenf** | | | **P49b** \|__\| **Abvenf** | | **Quabvenf**  \|__\|__\|,\|__\| piece | | | | | **P49c** \|__\| **Abdsenf** | |
| Fresh fish | | **P50a** \|__\|__\|,\|__\| **Pfagenf** | | | **P50b** \|__\| **Pfvenf** | | **Quapfvenf**  \|__\|__\|,\|__\| piece | | | | | **P50c** \|__\| **Pfdsenf** | |
| Dried fish | | **P51a** \|__\|__\|,\|__\| **Psagenf** | | | **P51b** \|__\| **Psvenf** | | **Quapsvenf**  \|__\|__\| piece | | | | | **P51c** \|__\| **Psdsenf** | |
| Shrimps | | **P52a** \|__\|__\|,\|__\| **Cragenf** | | | **P52b** \|__\| **Crvenf** | | **Quacrvenf**  \|__\|__\|,\|__\| piece | | | | | **P52c** \|__\| **Crdsenf** | |
| Broth of mealt/poultry/fish… | | **P53a** \|__\|__\|,\|__\| **Bviagenf** | | | **P53b** \|__\| **Bvivenf** | | **Quabvivenf**  \|__\|__\|,\|__\| glass | | | | | **P53c** \|__\| **Bvidsenf** | |
| Other products sea/freshwater**Pmenf** | | **P54a** \|__\|__\|,\|__\| **Pmagenf** | | | **P54b** \|__\| **Pmvenf** | | **Quapmvenf**  \|__\|__\|,\|__\| | | | | | **P54c** \|__\| **Pmdsenf** | |
| **Egg** | | **P55a** \|__\|__\|,\|__\| **Oagenf** | | | **P55b** \|__\| **Ovenf** | | **Quaovenf**  \|__\|__\|,\|__\| pieces | | | | | **P55c** \|__\| **Odsenf** | |
| **Vegetables and leaves** | | | | | | | | | | | | | |
| Carot | | **P56a** \|__\|__\|,\|__\| **Caagenf** | | | **P56b** \|__\| **Cavenf** | | **Quacavenf**  \|__\|__\|,\|__\| piece/ spoon | | | | | **P56c** \|__\| **Cadsenf** | |
| Cabbage | | **P57a** \|__\|__\|,\|__\| **Choagenf** | | | **P57b** \|__\| **Chovenf** | | **Quachovenf**  \|__\|__\|,\|__\| piece/ spoon | | | | | **P57c** \|__\| **Chodsenf** | |
| Pumpkin | | **P58a** \|__\|__\|,\|__\| **Ciagenf** | | | **P58b** \|__\| **Civenf** | | **Quacivenf**  \|__\|__\|,\|__\|piece/ spoon | | | | | **P58c** \|__\| **Cidsenf** | |
| Zucchini | | **P59a** \|__\|__\|,\|__\| **Coagenf** | | | **P59b** \|__\| **Covenf** | | **Quacovenf**  \|__\|__\|,\|__\| piece/ spoon | | | | | **P59c** \|__\| **Codsenf** | |
| Green bean | | **P60a** \|__\|__\|,\|__\| **Hvagenf** | | | **P60b** \|__\| **Hvvenf** | | **Quahvvenf**  \|__\|__\|,\|__\| piece/ spoon | | | | | **P60c** \|__\| **Hvdsenf** | |
| Onions | | **P61a** \|__\|__\|,\|__\| **Oiagenf** | | | **P61b** \|__\| **Oivenf** | | **Quaoivenf**  \|__\|__\|,\|__\| piece | | | | | **P61c** \|__\| **Oidsenf** | |
| Tomato | | **P62a** \|__\|__\|,\|__\| **Toagenf** | | | **P62b** \|__\| **Tovenf** | | **Quatovenf**  \|__\|__\|,\|__\| piece | | | | | **P62c** \|__\| **Todsenf** | |
| Other legumes **Lemenf** | | **P63a** \|__\|__\|,\|__\|  **Lemagenf** | | | **P63b** \|__\| **Lemvenf** | | **Qualemvenf**  \|__\|__\|,\|__\| piece/spoon | | | | | **P63c** \|__\| **Lemdsenf** | |
| Chinese cabbage | | **P64a** \|__\|__\|,\|__\| **Ccagenf** | | | **P64b** \|__\| **Ccvenf** | | **Quaccvenf**  \|__\|__\|,\|__\| spoon | | | | | **P64c** \|__\| **Ccdsenf** | |
| Anamamy | | **P65a** \|__\|__\|,\|__\| **Amagenf** | | | **P65b** \|__\| **Amvenf** | | **Quamvenf**  \|__\|__\|,\|__\| spoon | | | | | **P65c** \|__\| **Amdsenf** | |
| Anandrano | | **P66a** \|__\|__\|,\|__\| **Anagenf** | | | **P66b** \|__\| **Anvenf** | | **Quanvenf**  \|__\|__\|,\|__\| spoon | | | | | **P66c** \|__\| **Andsenf** | |
| Anatsinahy | | **P67a** \|__\|__\|,\|__\| **Atagenf** | | | **P67b** \|__\| **Atvenf** | | **Quatvenf**  \|__\|__\|,\|__\| spoon | | | | | **P67c** \|__\| **Atdsenf** | |
| Ravim-bomanga | | **P68a** \|__\|__\|,\|__\| **Rmagenf** | | | **P68b** \|__\| **Rmvenf** | | **Quarmvenf**  \|__\|__\|,\|__\| spoon | | | | | **P68c** \|__\| **Rmdsenf** | |
| Ravin-tsaosety | | **P69a** \|__\|__\|,\|__\| **Rsagenf** | | | **P69b** \|__\| **Rsvenf** | | **Quarsvenf**  \|__\|__\|,\|__\| spoon | | | | | **P69c** \|__\| **Rsdsenf** | |
| Ravitoto | | **P70a** \|__\|__\|,\|__\| **Rtagenf** | | | **P70b** \|__\| **Rtvenf** | | **Quartvenf**  \|__\|__\|,\|__\| spoon | | | | | **P70c** \|__\| **Rtdsenf** | |
| Broth of vegetables / leaves | | **P71a** \|__\|__\|,\|__\| **Blemagenf** | | | **P71b** \|__\| **Blemvenf** | | **Quablemvenf**  \|__\|__\|,\|__\| glass | | | | | **P71c** \|__\| **Blemdsenf** | |
| Other leaves **Brenf** | | **P72a** \|__\|__\|,\|__\| **Bragenf** | | | **P72b** \|__\| **Brvenf** | | **Quabrvenf**  \|__\|__\|,\|__\| spoon | | | | | **P72c** \|__\| **Brdsenf** | |
| **Fruits** | | | | | | | | | | | | | |
| Fruit juice | | **P73a** \|__\|__\|,\|__\| **Jusagenf** | | | **P73b** \|__\| **Jusvenf** | | **Quajusvenf**  \|__\|__\|,\|__\| glass | | | | | **P73c** \|__\| **Jusdsenf** | |
| Citrus | | **P74a** \|__\|__\|,\|__\| **Agragenf** | | | **P74b** \|__\| **Agrvenf** | | **Quagrvenf**  \|__\|__\|,\|__\| pieces | | | | | **P74c** \|__\| **Agrdsenf** | |
| Banana | | **P75a** \|__\|__\|,\|__\| **Banagenf** | | | **P75b** \|__\| **Banvenf** | | **Quabanvenf**  \|__\|__\| ,\|__\|pieces | | | | | **P75c** \|__\| **Bandsenf** | |
| Sugar cane | | **P76a** \|__\|__\|,\|__\| **Canagenf** | | | **P76b** \|__\| **Canvenf** | | **Quacanvenf**  \|__\|__\|,\|__\| pieces | | | | | **P76c** \|__\| **Candsenf** | |
| Sourspo | | **P77a** \|__\|__\|,\|__\| **Coragenf** | | | **P77b** \|__\| **Corvenf** | | **Quacorvenf**  \|__\|__\|,\|__\| pieces | | | | | **P77c** \|__\| **Cordsenf** | |
| Guava | | **P78a** \|__\|__\|,\|__\| **Goagenf** | | | **P78b** \|__\| **Govenf** | | **Quagovenf**  \|__\|__\|,\|__\| pièces | | | | | **P78c** \|__\| **Godsenf** | |
| Mango | | **P79a** \|__\|__\|,\|__\| **Magagenf** | | | **P79b** \|__\| **Magvenf** | | **Quamagvenf**  \|__\|__\|,\|__\| pieces | | | | | **P79c**\|__\| **Magdsenf** | |
| Papaya | | **P80a** \|__\|__\|,\|__\| **Papagenf** | | | **P80b** \|__\| **Papvenf** | | **Quapapvenf**  \|__\|__\|,\|__\| pieces | | | | | **P80c** \|__\| **Papdsenf** | |
| Other fruits **Fruitenf** | | **P81a** \|__\|__\|,\|__\| **Fruagenf** | | | **P81b** \|__\| **Fruvenf** | | **Quafruvenf**  \|__\|__\|,\|__\| pieces | | | | | **P81c** \|__\| **Frudsenf** | |
| **Oil and fat** | | | | | | | | | | | | | |
| Vegetal oil | | **P82a** \|__\|__\|,\|__\| **Huvagenf** | | | **P82b** \|__\| **Huvenf** | | | | | | | **P82c** \|__\| **Huvdsenf** | |
|  |  | - If the child consumed a flat added by vegetable oil yesterday, how many people consumed this meal?  - Quantity of oil added in family meal yesterday **Quahuv**  - Quantity of the dish consumed by the child **Platvenf** | | | | | | | | | | \|__\|__\|  \|__\|__\| spoon  \|__\|__\| bowl | |
| Animal fat | | **P83a** \|__\|__\|,\|__\| **Hanagenf** | | | **P83b** \|__\| **Hanvenf** | | **Quahanvenf**  \|__\|__\|,\|__\| spoon | | | | | **P83c** \|__\| **Handsenf** | |
| Others (to be precised) **Authugr** | | **P84a** \|__\|__\|,\|__\| **Hugragenf** | | | **P84b** \|__\| **Hugrvenf** | | **Quahugrvenf**  \|__\|__\|,\|__\| spoon | | | | | **P84c** \|__\| **Hugrdsenf** | |
| **Sweet products** | | | | | | | | | | | | | |
| Sweet/Sugar water | | **P85a** \|__\|__\|,\|__\| **Esagenf** | | | **P85b** \|__\| **Esvenf** | | **Quaesvenf**  \|__\|__\|,\|__\| glass | | | | | **P85c** \|__\| **Esdsenf** | |
| Tea, coffee | | **P86a** \|__\|__\|,\|__\| **Tcagenf** | | | **P86b** \|__\| **Tcvenf** | | **Quacvenf**  \|__\|__\|,\|__\| glass | | | | | **P86c** \|__\| **Tcdsenf** | |
| Honey | | **P87a** \|__\|__\|,\|__\| **Miagenf** | | | **P87b** \|__\| **Mivenf** | | **Quamivenf**  \|__\|__\|,\|__\|spoon | | | | | **P87c** \|__\| **Midsenf** | |
| Candy | | **P88a** \|__\|__\|,\|__\| **Bonagenf** | | | **P88b** \|__\| **Bonvenf** | | **Quabonvenf**  \|__\|__\|,\|__\| piece | | | | | **P88c** \|__\| **Bondsenf** | |
| **Water** | | **P89a** \|__\|__\|,\|__\| **Eagenf** | | | **P89b** \|__\| **Eavenf** | | **Quaeavenf**  \|__\|__\|,\|__\| glass | | | | | **P89c** \|__\| **Eadsenf** | |
| Other food **Aualim**  ___________ | | **P90a** \|__\|__\|,\|__\| **Aliagenf** | | | **P90b** \|__\| **Alivenf** | | **Qualivenf**  \|__\|__\|,\|__\| | | | | | **P90c** \|__\| **Alidsenf** | |
| ***Health practices*** | | | | | | | | | | | | | |
| **P91** | Does the child possesses a health, weighing or immunization record (1) Yes; (0) No ; (2) Nsp | | | | | | | | | | \|__\| **Carnet** | | |
| **P92a**  **P92b** | What is his/her birth weight (in kg)?  (1) Checked; (2) Said by the mother; (3) Unkown | | | | | | | | | | \|__\|__\|, \|__\|__\| kg  **Pnaiss**  \|__\| **Pverd** | | |
| **P93**  **P93a**  **P93b** | For this child, did you follow prenatal consultations? (1) Yes; (0) No ; (2) Nsp  If yes, how many times? (Note 99 if « do not know»)  - At the doctor or midwife  - At a traditional birth attendant | | | | | | | | | | \|__\| **Conspren**  \|__\|__\|  **Consaf**  \|__\|__\|  **Conmat** | | |
| **P94** | Where did the delivery take place? (1) hospital/Basic Health Center, (2) home (3) Others (to be specified) | | | | | | | | | | \|__\| **Lieuac**  ……………………**Lieuac_aut** | | |
| **P95a**  **P95b** | In the last 3 weeks, have you taken the child to a health center(1) Yes (0) No  If yes, why?  (1) Vaccination (2) Weighing (3) Disease  (4) Other (to be specified) | | | | | | | | | | \|__\| **Csant**  \|__\| **Rcsant**  …………………… **Aursant** | | |
| **P96a**  **P96b** | Has the child already received a dose of vitamin A like that in the last 6 months? *(Show the most common types of ampoules / capsule / syrups)*  (1) Yes ; (0) No ; (2) Not concerned (3) Don’t know  If yes, date of last intake of vitamin A | | | | | | | | | | \|__\| **Convita**  \|__\|__\|/\|__\|__\|/\|__\|__\| **Datvita** | | |
| **P97a**  **P97b** | In the past 6 months, has the child taken medicines for intestinal worms? (1) Yes ; (0) No ; (2) Not concerned (3) Don’t know  If yes, date of last intake of medicine against the intestinal worms | | | | | | | | | | \|__\| **Medver**  \|__\|__\|/\|__\|__\|/\|__\|__\| **Datmed** | | |
| **P98**  **P98a**  **P98b**  **P98c**  **P98d** | Was the child had have a food assistance? (1) Yes; (0) No ; (2) Don’t know  If yes, why? :  (1) Malnutrition (2) Poverty (3) Others (to be specified)  - What type(s) of food did you receive?  (1) Infant flour/porridge (2) milk in powder  (3) Other (to be specified)  - Date of the beginning of the food assistance  - Date of the end of the food assistance | | | | | | | | | | \|__\| **Aidalim**  \|__\| **Raidalim**  \|__\| **Talirec**  ……………………… **Aualirec**  \|__\|__\|/\|__\|__\|/\|__\|__\| **Datdeb**  \|__\|__\|/\|__\|__\|/\|__\|__\| **Datfin** | | |
| **P99**  **P99a**  **P99b**  **P99c**  **P99d** | Was the child taken care in a center of nutritional recovery? (1) Oui ; (0) Non ; (2) Don’t know  If yes, why? (1) Affected by malnutrition  (2) Others (to be specified)  - What type(s) of food did you receive?  (1) Plumpy nut/immunit  (2) Other (to be specified)  - Date of the beginning of the care  - Date of the end of the care | | | | | | | | | | \|__\| **Pricren**  \|__\| **Rpricren**  \|__\| **Alimrec**  …………………...**Aualimrec**  \|__\|__\|/\|__\|__\|/\|__\|__\| **Datdpr**  \|__\|__\|/\|__\|__\|/\|__\|__\| **Datfpr** | | |
| *Have the following vaccines been done?* | | | | | | | | | | | | | |
| Vaccin | | | | (1) Yes (0) No | | | | | (1) Checked; (2) Declared | | | | |
| BCG | | | | **P100a** \|__\| **Vbcg** | | | | | **P100b**  \|__\| **Bcgvd** | | | | |
| Polio O | | | | **P101a** \|__\| **Vpolioa** | | | | | **P101b** \|__\| **Polioavd** | | | | |
| DTCHB 1 | | | | **P102a** \|__\| **Dtchba** | | | | | **P102b** \|__\| **Dtchbavd** | | | | |
| Polio 1 | | | | **P103a** \|__\| **Poliob** | | | | | **P103b** \|__\| **Poliobvd** | | | | |
| PCV10 1 | | | | **P104a** \|__\| **Pcva** | | | | | **P104b** \|__\| **Pcvavd** | | | | |
| DTCHB 2 | | | | **P105a**  \|__\| **Dtchbb** | | | | | **P105b** \|__\| **Dtchbbvd** | | | | |
| Polio 2 | | | | **P106a** \|__\| **Polioc** | | | | | **P106b** \|__\| **Poliocvd** | | | | |
| PCV10 2 | | | | **P107a** \|__\| **Pcvb** | | | | | **P107b** \|__\| **Pcvbvd** | | | | |
| DTCHB 3 | | | | **P108a**  \|__\| **Dtchbc** | | | | | **P108b** \|__\| **Dtchbcvd** | | | | |
| Polio 3 | | | | **P109a** \|__\| **Poliod** | | | | | **P109b** \|__\| **Poliodvd** | | | | |
| PCV10 3 | | | | **P110a**  \|__\| **Pcvc** | | | | | **P110b** \|__\| **Pcvcvd** | | | | |
| VAR | | | | **P111a**  \|__\| **Var** | | | | | **P111b** \|__\| **Varvd** | | | | |
| Vaccine anti-rotavirus :  Dose 1  Dose 2 | | | | **P112a** \|__\| **Vrotvirus 1**  **P113a** \|__\| **Vrotvirus 2** | | | | | **P112b** \|__\| **Vrotvirusvd 1**  **P113b** \|__\| **Vrotvirusvd 2** | | | | |
| *Symptoms presented by the child* | | | | | | | | | | | | | |
| List of the symptoms | | | | | Presence of symptoms at time of investigation  (1)Yes (0) No | | | | Diseases or symptoms arisen during 14 days prior the investigation (1) Yes (0) No | | | | |
| Fever | | | | | **P114a** \|__\| **Fievreme** | | | | **P114b** \|__\| **Fievrede** | | | | |
| Cough | | | | | **P115a**  \|__\| **Touxme** | | | | **P115b** \|__\| **Touxde** | | | | |
| Anorexia | | | | | **P116a** \|__\| **Anorexme** | | | | **P116b** \|__\| **Anorexde** | | | | |
| Runny nose | | | | | **P117a** \|__\| **Ecnasme** | | | | **P117b** \|__\| **Ecnasde** | | | | |
| Vomiting | | | | | **P118a** \|__\| **Vomime** | | | | **P118b** \|__\| **Vomide** | | | | |
| Abdominal pain | | | | | **P119a**  \|__\| **Dolabme** | | | | **P119b** \|__\| **Dolabde** | | | | |
| Diarrhea | | | | | **P120a** \|__\| **Diarrheme** | | | | **P120b** \|__\| **Diarrhede** | | | | |
| In case of diarrhea  (**P120a ou P120b =1**):  - Number of stools emitted by the child for one day?  - Consistency? :  - (1) Loose  - (2) Liquids  - (3) Pasty | | | | | **P120c** \|__\| **Nbselleme**  **P120d** \|__\| **Consistme** | | | | **P120e** \|__\| **Nbsellede**  **P120f** \|__\| **Consistde** | | | | |
| Conjunctivitis | | | | | **P121a** \|__\| **Conjome** | | | | **P121b** \|__\| **Conjode** | | | | |
| Earache | | | | | **P122a** \|__\| **Otalgime** | | | | **P122b** \|__\| **Otalgide** | | | | |
| Dyspnea | | | | | **P123a** \|__\| **Dyspneme** | | | | **P123b** \|__\| **Dyspnede** | | | | |
| Skin rashes | | | | | **P124a** \|__\| **Ercutme** | | | | **P124b** \|__\| **Ercutde** | | | | |
| Others diseases  **Autmala** | | | | | **P125a** \|__\| **Autmalme** | | | | **P125b** \|__\| **Autmalde** | | | | |
| Did the child take any medication? (1) Yes (0) No  If yes, what medicine did he take?  - Medicine1:  - Medicine 2:  - Medicine 3:  - Medicine 4:  - Medicine 5:  - Medicine 6 : | | | | | **P126a** \|__\| **Medprisme**  ……………. **Medme 1**  ……………. **Medme 2**  ……………. **Medme 3**  ……………. **Medme 4**  …………….. **Medme 5**  …………….. **Medme 6** | | | | **P126b** \|__\| **Medprisde**  ……………. **Medde 1**  ……………. **Medde 2**  ……………. **Medde 3**  ……………. **Medde 4**  …………….. **Medde 5**  …………….. **Medde 6** | | | | |
