# Supplementary material for: The importance of public health, poverty reduction programs and women’s empowerment in the reduction of child stunting in rural areas of Moramanga and Morondava, Madagascar
Source: PLoS One. 2017 Oct 18;12(10):e0186493. doi: 10.1371/journal.pone.0186493 (PMC5646813; doi:10.1371/journal.pone.0186493)
Supplement: S3 Text — (DOCX) [file pone.0186493.s004.docx]

**Questionnaire on the household and mother/person in charge of the child**

| **Household identification** | | | | | | | | | |
| --- | --- | --- | --- | --- | --- | --- | --- | --- | --- |
| **M1** | District | | | | | \|__\|__\| **District** | | | |
| **M2** | Commune | | | | | \|__\|__\| idcommune | | | |
| **M3** | Fokontany | | | | | \|__\|__\| idfkt | | | |
| **M4** | Hamlet | | | | | \|__\|__\| idhameau | | | |
| **M5** | Home | | | | | \|__\|__\|__\| **Foyer** | | | |
| **M6** | Adress | | | | | mais_adr | | | |
| **M7** | Household identification: \|__\|__\|__\|__\|__\|__\|__\|__\|__\|__\|__\|__\|__\| idmenage | | | | | | | | |
| **M8** | Investigation’s date | | | | | \|__\|__\|/\|__\|__\|/\|__\|__\| **Datenquete** | | | |
| **M9** | Interviewer code | | | | | \|__\|__\| enqueteur | | | |
| **Characteristics of the mother or the person who usually takes care of the child at the house (POHE)** | | | | | | | | |  |
| **M10** | | Family ties of the respondent with the child: (1) Mother (2) POHE  If 2, who is the POHE? (1) Father (2) Sister/Brother  (3) Grandparent/Aunt (4) Nanny (5) Other (to be specified) : | | | | | \|__\| **Lienrep**  \|__\| **Pohe**  ………….**Pohe_aut** | |  |
| **M11** | | Name and first names of the respondent: | | | | | **Nomer** | |  |
| **M12a** | | Date of birth : | | | | | \|__\|__\|/\|__\|__\|/\|__\|__\| **Datmer** | |  |
| **M12b** | | Age (in year) : | | | | | \|__\|__\| **Agmer** | |  |
| **M13a**  **M13b** | | - Who is the head of the household? (Family ties with the child) (1) Father (2) Mother (3) Sister/Brother  (4) Grandparent (5) Other (to be specified) :  - Note the gender of the head of the household  (1) Male (2) Female | | | | | \|__\| **Chefmen**  ……….**Autchefmen**  \|__\| **Sechefmen** | |  |
| **M14** | | Level of maximum schooling: (*what is the most advanced reached class*?)  (1) Never schooled  (2) Primary level  (3) Secondary level I  (4) Secondary level II  (5) Upper level  (6) Don’t know | | | | | \|__\| scolar_niv | |  |
| **M15** | | Marital status :  (1).Never married  (2).Monogamous married  (3).Polygamous married  (4).Common-law relationship (common life without marriage)  (5).Divorced  (6).Separated  (7).Window | | | | | \|__\| statmat | |  |
| **M16** | | Current job:  (1).Activity with current income  (2).Unemployed person  (3).Housewife  (4).Student  (5).Retired  (6). Not capable: disabled, chronic sick person  (7).Other (to be specified) | | | | | \|__\| emploi  ………….. emploi_aut | |  |
| **M17** | | Status of main activity: *(What is your main status to earn money?)*  (1). Permanent employee of a private enterprise  (2). Seasonal employee of a private enterprise  (3). Patron of a private company  (4). Employee of a public institution  (5). Liberal/independent (lawyer, medical practitioner...)  (6) .Artisan  (7).Domestic workers/household staff (familial help)  (8). Informal activity (sale of street, participation in the family activity …)  (9). None  (10).Other, to be specified | | | | | \|__\|__\| act_stat  …………. act_stat_aut | |  |
| **M18** | | Activity area :*( In what field is this activity?)*  (1).Agriculture  (2).Commerce and services  (3).Craft and small company  (4).Heavy industry/ Mining industry  (5).Big companies  (6).Public function (expect education and health)  (7).Health  (8).Education  (9).Other (to be specified) | | | | | \|__\| act_dom  ……………. act_aut | |  |
| **M19** | | If mother, number of pregnancies which you had *(by counting miscarriages and child being investigated) (99 if not concerned)* | | | | | \|__\|__\| **nbgros** | |  |
| **M20** | | Number of children still living if mother or number of dependent children if *POHE* | | | | | \|__\|__\| **Nbenf** | |  |
| **M21a** | | Birth rank of the child in question | | | | | \|__\|__\| **Ranenf** | |  |
| **M21b** | | How old is the elder child of the weighed child (in months) | | | | | \|__\|__\|,\|__\| **Intagenf** | |  |
| **M22** | | Are you currently pregnant? (1) Yes (0) No | | | | | \|__\| **Enceinte** | |  |
| *Satisfaction of mother / POHE in various field* | | | | | | | | |  |
| **M23** | | *Attitudes towards the domestic violence:*  Sometimes a husband is upset or angry because of certain things as his wife made, in your opinion, is it justified for a husband to strike or beat his wife in the following situations? If she :  - Goes out without saying him(1) Yes (0) No (3) No idea  - Neglects the children (1) Yes (0) No (3) No idea  - Argues with him (1) Yes (0) No (3) No idea  - Refuses to have sex with him (1) Yes (0) No (3) No idea  - Burns the food (1) Yes (0) No (3) No idea | | | | | \|__\| **Sort**  \|__\| **Neglige**  \|__\| **Argum**  \|__\| **Refrap**  \|__\| **Brulnou** | |  |
| **M24** | | To what extent you are satisfied by the way your husband treats you? (Show the photos)  (1) Very satisfied  (2) A little satisfied  (3) Neither satisfied nor dissatisfied  (4) A little dissatisfied  (5) Very dissatisfied | | | | | \|__\| **Traitmari** | |  |
| **M25** | | To what extent you are satisfied by your health?  (1) Very satisfied  (2) A little satisfied  (3) Neither satisfied nor dissatisfied  (4) A little dissatisfied  (5) Very dissatisfied | | | | | \|__\| **Sante** | |  |
| **M26** | | To what extent you are satisfied by your place where you live? *(If necessary, explain to her that the question refers to the environment of life, including the district/neighborhood and the house)*  (1) Very satisfied  (2) A little satisfied  (3) Neither satisfied nor dissatisfied  (4) A little dissatisfied  (5) Very dissatisfied | | | | | \|__\| **Endhab** | |  |
| **M27** | | To what extent you are satisfied by your life, generally?  (1) Very satisfied  (2) A little satisfied  (3) Neither satisfied nor dissatisfied  (4) A little dissatisfied  (5) Very dissatisfied | | | | | \|__\| **Viegen** | |  |
| **M28** | | To what extent you are satisfied by your income?  (1) Very satisfied  (2) A little satisfied  (3) Neither satisfied nor dissatisfied  (4) A little dissatisfied  (5) Very dissatisfied *(If the respondent has answers that she has no income, surround "0* | | | | | \|__\| **Revenu** | |  |
| **M29** | | Are you satisfied by the way your children treat you? (Show the photos)  (1) Very satisfied  (2) A little satisfied  (3) Neither satisfied nor dissatisfied  (4) A little dissatisfied  (5) Very dissatisfied | | | | | \|__\| **Traitenf** | |  |
| **M30** | | Are you happy in your married / family life?  (1) Very happy  (2) A little happy  (3) Neither happy nor unhappy  (4) A little unhappy  (5) Very unfortunate | | | | | \|__\| **Vieconj** | |  |
| **Characteristics of the household** | | | | | | | | |  |
| *Household information* | | | | | | | | |  |
| **M31** | | How many people live permanently in the household? (Number of the absent or present residents) | | | | | \|__\|__\| **Perstoit** | |  |
| **M32** | | Number of children under 5 years living permanently in the household (housework) | | | | | \|__\|__\| **Nbenf** | |  |
| *Habitat characteristics* | | | | | | | | |  |
| **M33** | | Occupation status:  (1) Owner  (2) Tenant  (3) Accommodated for free (by employer or family)  (4) Other (to be specified) | | | | | \|__\| bail  ……….. bail_aut | |  |
| **M34** | | Type of the housing environment:  (1) Apartment  (2) Individual house  (3) Rooms in a house with multiple housings  (Kitchen or common shower-room)  (4) Shed/Cottage  (5) Other (to be specified) | | | | | \|__\| hab_typ    ……….. hab_aut | |  |
| **M35** | | Number of livable rooms (*How many livable rooms do you have? (Except toilet, shower-room and kitchen )* | | | | | \|__\|__\| piece_ nb | |  |
| **M36** | | Type of wall (*what is the main material of your wall?)*  (1) Brick  (2) Stone  (3) Concrete  (4) Sheet metal  (5) Bought wood (board)  (6) Ravinala/falafa(kind of leaves in rural area of Madagascar)  (7) Bamboo/volo  (8) Rush  (9) Soil /Land  (10) Collected wood  (11) Other (to be specified) | | | | | \|__\| murs  ……….. murs_aut | |  |
| **M37** | | Type of roof (What is the main material of the roof of your house?)  (1) Concrete  (2) Tile(blow)  (3) Sheet steel  (4) Bought wood (board)  (5) Thatch  (6) Ravinala  (7) Other plants  (8) Other (to be specified ) | | | | | \|__\| toit  ……….. toit_aut | |  |
| **M38** | | Type of the floor:  (1) Tiles  (2) Parquet  (3) Cement  (4) Vinyl (balatum)  (5) Bought wooden board  (6) Ground /sand  (7) Plants or collected wood(ravinala)  (8) Other (to be specified) | | | | | \|__\| sol  ………. sol_aut | |  |
| **M39** | | Lighting: (What is the usual lighting mode?)  (1) Electricity  (2) Lantern oil  (3) Candlelight  (4) Others (to be specified )  (5) No lighting | | | | | \|__\| **eclair**  …………**.eclair_aut** | |  |
| **M40** | | Fuel (kitchen): (*What is the usual way to cook food every day?)*  (1) Gas bottle  (2) Electricity  (3) Petroleum  (4) Bought charcoal  (5) Wood  (6) Dung  (7) Others (to be specified) | | | | | \|__\| combust  ……….combust_aut | |  |
| *Patrimoines* | | | | | | | | |  |
| **M41a**  **M41b**  **M41c**  **M41d**  **M41e**  **M41f**  **M41g**  **M41h**  **M41i**  **M41j**  **M41k**  **M41l**  **M41m**  **M42a**  **M42b**  **M43a**  **M43b**  **M43c**  **M43d**  **M44a**  **M44b**  **M44c**  **M44d**  **M44e**  **M44f**  **M45a**  **M45b**  **M45c**  **M45d**  **M45e**  **M46a**  **M46b**  **M46c**  **M46d**  **M46e**  **M47**  **M47a**  **M47b** | | Does the household own? (note the number)  - Radio  - Television  - Computer  - Internet access  - Fixed line telephone  - Mobile phone  - Refrigerator  - Sewing machine  - Car  - Motorcycle / Motorcycle  - Truck  - Tractor  - Other motor vehicle: specify **Vehm**……………..  - Bicycle  - Cart with zebus  *Pets :*  Dogs  Cats  Birds  Others (to be specified)  - *Farmyard animals :*  Chikens  Goose  Ducks  Guinea fowl  Rabbits  Others (to be specified)………………………….  - *Animals of breeding:*  Cows / Zebu  Porks  Sheeps  Goats  Others (to be specified) ………………………..  - *Vegetable gardens/Orchards :*  Fruits  Vegetables (zucchinis, leeks, cabbage,…)  Tubers (potatoes, sweet potatoes, yams, ...)  Legumes (beans, peas ...)  Others (to be specified)…………………………..  - Rice fields  - Rented rice fields  - Houses or rented apartments | | | | | \|__\|__\| radio  \|__\|__\| tv  \|__\|__\| ordi  \|__\|__\| internet  \|__\|__\| tel_fix  \|__\|__\| tel_mob_nb  \|__\|__\| frigo  \|__\|__\| mach_coud  \|__\|__\| voiture  \|__\|__\| moto  \|__\|__\| camion  \|__\|__\| tract  \|__\|__\| vehic_aut  \|__\|__\| velo  \|__\|__\| charette  \|__\|__\| chien  \|__\|__\| chat  \|__\|__\| oiseau  \|__\|__\| dom_aut_nb  \|__\|__\| poul  \|__\|__\| oie  \|__\|__\| canard  \|__\|__\| pintad  \|__\|__\| lapin  \|__\|__\| basc_aut_nb  \|__\|__\| zebu  \|__\|__\| porc  \|__\|__\| mouton  \|__\|__\| chevre  \|__\|__\| elv_au_nb  \|__\|__\| fruit  \|__\|__\| legume  \|__\|__\| tuberc  \|__\|__\| legumin  \|__\|__\| potag_ aut  \|__\|__\| riziere  \|__\|__\| riziere_loc  \|__\|__\| log_loc | |  |
| *Hygiene of the household* | | | | | | | | |  |
| **M48** | | Modality of evacuation of the garbage/domestic waste: (*How do you usually get rid of your garbage?)*  (1) Collection of the city  (2) Deposit in a pit of the city  (3) Deposit on the road  (4) Incineration on site  (5) No fixed evacuation mode  (6) Other (to be specified) | | | | | \|__\| ordur_evac  ……….. ….ordur_aut | |  |
| **M49** | | Toilet used by the household  (1) Toilets + flush, private interiors  (2) Toilets + flush, common interiors  (3) In-ground individual latrine outdoor  (4) In-ground common latrine outdoor  (5) In the nature  (6) Other (to be specified) | | | | | \|__\| latrine  …………… latrine_aut | |  |
| **M50** | | Local to wash *(Where do the family members go to wash himself most of the time?):*  (1) Interior, specific room (shower room)  (2) Interior without specific room (kitchen...)  (3) Exterior specific shelter  (4) Exterior without shelter (in courtyard)  (5) River/ Puddle  (6) Other, to precise | | | | | \|__\| douche  ………… douche_aut | |  |
| **M51** | | Drinking-water supply: *(Where do you usually look for drinking water?)*  (1) Purchased (eau vive,…)  (2) Private faucet  (3) A public standpipe  (4) Private well  (5) Collective well  (6) Private drilling (makiplast…)  (7) Public pump of undetermined origin  (8) Watercourse/ Source  (9) Puddle/ Collection of rainwater  (10) Other (to be specified) | | | | | \|__\| eau_bois  …………………….eau_bois_aut | |  |
| **M52** | | Distance between the house and the drinking water supply point: (*How long do we go on foot to get drinking water?)*  (1) Less than 5 minutes  (2) Between 5 and 10 minutes  (3) Between 10 and 15 minutes  (4) Between 15 and 30 minutes  (5) More than 30 minutes | | | | | \|__\| eau_dist | |  |
| **M53**  **M53a** | | Modality to store drinking water: *(Where do you usually store drinking water?)*  (1) Outside  (2) Inside the house  (3) Do not store  For those who store drinking water, is the storage container protected (covered or closed with a plug)?  (0) No (1) Yes | | | | | \|__\| **eau_stock**  \|__\| **Constock** | |  |
| **M54**  **M55** | | Treatment of drinking water*: (Do you treat drinking water before drinking?)* (1) Yes (0) No  If yes, What treatment?  (1) Filtering(2) Boiling (3) Simple decantation  (4) Add products (6) Other (to be specified) | | | | | \|__\| **eau_tt**  \|__\| **Natrait**  ……….**Natrait_aut** | |  |
| **M56** | | Where do you cook?  (1) In a specific room inside  (2) In a common room inside  (3) Outside in a shelter  (4) Outside in the court or the street  (5) Other (to be specified) | | | | | \|__\| cuisine  ……… cuisine_aut | |  |
| *Household food* | | | | | | | | |  |
| **M57** | | Yesterday, did the mother / POHE eat at home?  (1) Yes (0) No | | | | | \|__\| **Consmais** | |  |
| **M58** | | How much did you spend in *Ariary* for the meal preparation of the family member during the day of yesterday?(Note 99999 if the respondent do not know) | | | | | \|__\|__\|__\|__\|__\|  **alim_budg** | |  |
| **M59a**  **M59b**  **M59c**  **M59d**  **M59e** | | Yesterday, did the mother take?  *Breakfast*: (1) Yes (0) No  *Snack/morning* : (1) Yes (0) No  *Lunch* : (1) Yes (0) No  *Snack/afternoon* : (1) Yes (0) No  *Dinner* : (1) Yes (0) No | | | | | \|__\| **Pdejmer**  \|__\| **Gmatmer**  \|__\| **Dejmer**  \|__\| **Gapremer**  \|__\| **Dinmer** | |  |
| *Food practices of the mother/household* | | | | | | | | |  |
| Food | | | Yesterday, did the mother eat? (1) Yes (0) No | If yes, how many times yesterday? | Did she consume the last week?  (1) Yes (0) No | | | If yes, how many of the day during the last week? (Rate from 1 to 7) |  |
| *Cereals* (Rice, wheat, corn, sorghum, pasta, bread, biscuits, flour) | | | **M60a** \|__\|  **Cervmer** | **M60b** \|__\|  **Cernbmer** | **M60c** \|__\|  **Cerdsmer** | | | **M60d** \|__\|  **Cernjmer** |  |
| *Roots and tubers* (Sweet potatoes , taro, cassava, potatoes) | | | **M61a** \|__\|  **Rtvmer** | **M61b** \|__\| **Rtnbmer** | **M61c** \|__\| **Rtdsmer** | | | **M61d** \|__\| **Rtnjmer** |  |
| *Legumes* (Beans, Cowpeas, Peas, Lentils, ...) | | | **M62a** \|__\|  **Legvmer** | **M62b** \|__\|  **Legnbmer** | **M62c** \|__\|  **Legdsmer** | | | **M62d** \|__\|  **Legnjmer** |  |
| *Milk / dairy products* (yogurt, cheese, milk powder ...) | | | **M63a** \|__\|  **Lvmer** | **M63b** \|__\|  **Lnbmer** | **M63c** \|__\|  **Ldsmer** | | | **M63d** \|__\|  **Lnjmer** |  |
| *Meat, Poultry, Offal* (beef, pork, chicken, liver, ...) | | | **M64a** \|__\|  **Vivmer** | **M64b** \|__\|  **Vinbmer** | **M64c** \|__\|  **Vidsmer** | | | **M64d** \|__\|  **Vinjmer** |  |
| *Fish and seafood* (fresh or dried) | | | **M65a** \|__\|  **Povmer** | **M65b** \|__\|  **Ponbmer** | **M65c** \|__\|  **Podsmer** | | | **M65d** \|__\|  **Ponjmer** |  |
| *Eggs* | | | **M66a** \|__\|  **Ovmer** | **M66b** \|__\|  **Onbmer** | **M66c** \|__\|  **Odsmer** | | | **M66d** \|__\|  **Onjmer** |  |
| *Fruits* (Apple, pineapple, banana, avocado, soursop, lychee, mango, guava, papaya, kaki ...) | | | **M67a** \|__\|  **Fruvmer** | **M67b** \|__\|  **Frunbmer** | **M67c** \|__\|  **Frudsmer** | | | **M67d** \|__\|  **Frunjmer** |  |
| *Vegetables and breeds* (Tomato, zucchini, green bean, cabbage, carrot, turnip, Chinese cabbage …) | | | **M68a** \|__\|  **Lemvmer** | **M68b** \|__\|  **Lemnbmer** | **M68c** \|__\|  **Lemdsmer** | | | **M68d** \|__\|  **Lemnjmer** |  |
| *Other (to be specified)*  **Aualimer**  ………………… | | | **M69a** \|__\|  **Alivmer** | **M69b** \|__\|  **Alinbmer** | **M69c** \|__\|  **Alidsmer** | | | **M69d** \|__\|  **Alinjmer** |  |
