# Supplementary material for: The importance of public health, poverty reduction programs and women’s empowerment in the reduction of child stunting in rural areas of Moramanga and Morondava, Madagascar
Source: PLoS One. 2017 Oct 18;12(10):e0186493. doi: 10.1371/journal.pone.0186493 (PMC5646813; doi:10.1371/journal.pone.0186493)
Supplement: S4 Text — (DOCX) [file pone.0186493.s005.docx]

**Questionnaire sur la mère ou de la personne en charge de l’enfant**

| **Identification du ménage** | | | | | | | | | |
| --- | --- | --- | --- | --- | --- | --- | --- | --- | --- |
| **M1** | District | | | | | \|__\|__\| **District** | | | |
| **M2** | Commune | | | | | \|__\|__\| idcommune | | | |
| **M3** | Fokontany | | | | | \|__\|__\| idfkt | | | |
| **M4** | Hameau | | | | | \|__\|__\| idhameau | | | |
| **M5** | Foyer | | | | | \|__\|__\|__\| **Foyer** | | | |
| **M6** | Adresse | | | | | mais_adr | | | |
| **M7** | Identification du ménage : \|__\|__\|__\|__\|__\|__\|__\|__\|__\|__\|__\|__\|__\| idmenage | | | | | | | | |
| **M8** | Date d’enquête | | | | | \|__\|__\|/\|__\|__\|/\|__\|__\| **Datenquete** | | | |
| **M9** | Code enquêteur | | | | | \|__\|__\| enqueteur | | | |
| **Caractéristiques de la mère ou de la personne qui s’occupe habituellement de l’enfant à la maison (POHE)** | | | | | | | | |  |
| **M10** | | Lien de parenté du repondant avec l’enfant :  (1) Mère (2) POHE  Si 2, qui est la POHE ? (1) Père (2) Sœur/Frère  (3) Grand parent/Tante (4) Nounou (5) Autre (à préciser) : | | | | | \|__\| **Lienrep**  \|__\| **Pohe**  ………….**Pohe_aut** | |  |
| **M11** | | Nom et prénoms du repondant : | | | | | **Nomer** | |  |
| **M12a** | | Date de naissance : | | | | | \|__\|__\|/\|__\|__\|/\|__\|__\| **Datmer** | |  |
| **M12b** | | Age (en année) : | | | | | \|__\|__\| **Agmer** | |  |
| **M13a**  **M13b** | | - Qui est le chef du ménage ? (Lien de parenté avec l’enfant) (1) Père (2) Mère (3) Sœur/Frère  (4) Grand parent (5) Autre (à préciser)  - Noter le sexe du chef de ménage  (1) Masculin (2) Féminin | | | | | \|__\| **Chefmen**  ……….**Autchefmen**  \|__\| **Sechefmen** | |  |
| **M14** | | Niveau de scolarisation maximum :  *(Quelle est la classe la plus avancée atteinte ?)*  (1) Jamais scolarisé  (2) Niveau primaire  (3) Niveau secondaire I  (4) Niveau secondaire II  (5) Niveau supérieur  (6) nsp | | | | | \|__\| scolar_niv | |  |
| **M15** | | Statut matrimonial :  (1).Jamais mariée  (2).Mariée monogame  (3).Mariée polygame  (4).Union libre (vie commune sans mariage)  (5).Divorcée  (6).Séparée  (7).Veuve | | | | | \|__\| statmat | |  |
| **M16** | | Emploi actuel:  (1).Activité avec revenu en cours  (2).Chômeur  (3).Femme au foyer  (4).Elève/étudiant  (5).Retraité  (6).Non apte : handicapé, malade chronique  (7).Autre (à préciser) | | | | | \|__\| emploi  ………….. emploi_aut | |  |
| **M17** | | Statut d’activité principale :(*Quel est votre statut principal pour gagner de l’argent ?)*  (1).Salarié permanent entreprise privée  (2).Salarié saisonnier d’une entreprise privée  (3).Patron d’une entreprise privée  (4).Salarié d’une institution publique  (5). Libéral/indépendant (avocat, médecin en cabinet, ...)  (6).Artisan  (7).Personnel de maison (aide familiale)  (8).Activité informelle (vente de rue, portage, participation à l’activité familiale…)  (9).Aucune  (10).Autre, préciser | | | | | \|__\|__\| act_stat  …………. act_stat_aut | |  |
| **M18** | | Domaine d’activité :*(Dans quel domaine est cette activité ?)*  (1).Agriculture  (2).Commerce et services  (3).Artisanat et petite entreprise  (4).Industrie lourde/ Industrie minière  (5).Grosses entreprises  (6).Fonction Publique (hors éducation et santé)  (7).Santé  (8).Education  (9).Autre (préciser) | | | | | \|__\| act_dom  ……………. act_aut | |  |
| **M19** | | *Si mère,* nombre de grossesses que vous avez eues *(en comptant les fausses couches et l’enfant enquêté) (99 si non concerné)* | | | | | \|__\|__\| **nbgros** | |  |
| **M20** | | Nombre d’enfants encore vivants si *mère* ou nombre d’enfants à charge pour *POHE* | | | | | \|__\|__\| **Nbenf** | |  |
| **M21a** | | Rang de naissance de l’enfant en question | | | | | \|__\|__\| **Ranenf** | |  |
| **M21b** | | Quel âge a l’enfant aîné de l’enfant pesé (en mois) | | | | | \|__\|__\|,\|__\| **Intagenf** | |  |
| **M22** | | Etes-vous enceinte actuellement ? (1) Oui (0) Non | | | | | \|__\| **Enceinte** | |  |
| *Satisfaction de la mère/POHE dans différents domaines* | | | | | | | | |  |
| **M23** | | *Attitudes vis-à-vis de la violence domestique* :  Parfois un mari est contrarié ou en colère à cause de certaines choses que sa femme a fait, à votre avis, est-il justifié qu’un mari frappe ou batte sa femme dans les situations suivantes? Si elle :  - Sort sans lui dire (1) Oui (0) Non (3) aucune idée  - Néglige les enfants (1) Oui (0) Non (3) aucune idée  - Argumente avec lui (1) Oui (0) Non (3) aucune idée  - Refuse d’avoir de rapport sexuel avec lui (1) Oui (0) Non (3) aucune idée  - Brule la nourriture (1) Oui (0) Non (3) aucune idée | | | | | \|__\| **Sort**  \|__\| **Neglige**  \|__\| **Argum**  \|__\| **Refrap**  \|__\| **Brulnou** | |  |
| **M24** | | Dans quelle mesure êtes-vous satisfaites de la façon dont votre mari vous traite? (montrer les photos)  (1) Très satisfaite  (2) Quelque peu satisfaite  (3) Ni satisfaite ni insatisfaite  (4) Quelque peu insatisfaite  (5) Très insatisfaite | | | | | \|__\| **Traitmari** | |  |
| **M25** | | Dans quelle mesure êtes-vous satisfaites de votre santé ?  (1) Très satisfaite  (2) Quelque peu satisfaite  (3) Ni satisfaite ni insatisfaite  (4) Quelque peu insatisfaite  (5) Très insatisfaite | | | | | \|__\| **Sante** | |  |
| **M26** | | Dans quelle mesure êtes-vous satisfaites de votre l’endroit où vous habitez? *(Si besoin, expliquez-lui que la question se réfère à l’environnement de vie, y compris le quartier et l’habitation.)*  (1) Très satisfaite  (2) Quelque peu satisfaite  (3) Ni satisfaite ni insatisfaite  (4) Quelque peu insatisfaite  (5) Très insatisfaite | | | | | \|__\| **Endhab** | |  |
| **M27** | | Dans quelle mesure êtes-vous satisfaites de votre vie, en générale?  (1) Très satisfaite  (2) Quelque peu satisfaite  (3) Ni satisfaite ni insatisfaite  (4) Quelque peu insatisfaite  (5) Très insatisfaite | | | | | \|__\| **Viegen** | |  |
| **M28** | | Dans quelle mesure êtes-vous satisfaites de votre revenu?  (1) Très satisfaite  (2) Quelque peu satisfaite  (3) Ni satisfaite ni insatisfaite  (4) Quelque peu insatisfaite  (5) Très insatisfaite  *(Si le répondant a répond qu’elle n’a pas de revenu, entourez “0”)* | | | | | \|__\| **Revenu** | |  |
| **M29** | | Etes-vous satisfaites de la façon dont vos enfants vous traitent? (montrer les photos)  (1) Très satisfaites  (2) Quelque peu satisfaite  (3) Ni satisfaite ni insatisfaite  (4) Quelque peu insatisfaite  (5) Très insatisfaite | | | | | \|__\| **Traitenf** | |  |
| **M30** | | Etes-vous heureuse dans votre vie conjugale/familiale ? (montrer les photos)  (1) Très heureuse  (2) Quelque peu heureuse  (3) Ni heureuse ni malheureuse  (4) Quelque peu malheureuse  (5) Très malheureuse | | | | | \|__\| **Vieconj** | |  |
| **Caractéristiques du ménage** | | | | | | | | |  |
| *Information sur le ménage* | | | | | | | | |  |
| **M31** | | Combien de personnes vivent en permanence sous le toit ? (nombre des résidents absents ou présents) | | | | | \|__\|__\| **Perstoit** | |  |
| **M32** | | Nombre d’enfants de moins de 5 ans vivant en permanence dans le ménage | | | | | \|__\|__\| **Nbenf** | |  |
| *Caractéristiques de l’habitat* | | | | | | | | |  |
| **M33** | | Statut d’occupation :  (1) Propriétaire  (2) Locataire  (3) Logé à titre gratuit (par employeur ou famille)  (4) Autre (à préciser) | | | | | \|__\| bail  ……….. bail_aut | |  |
| **M34** | | Type de l’habitat :  (1) Appartement  (2) Maison individuelle  (3) Pièces dans une maison avec plusieurs ménages  (cuisine ou salle d’eau commune)  (4) Cabanon  (5) Autres (préciser) | | | | | \|__\| hab_typ    ……….. hab_aut | |  |
| **M35** | | Nombre de pièces habitables (*avez-vous combien de pièces habitable? (En dehors des toilettes, salle d’eau et cuisine)* | | | | | \|__\|__\| piece_ nb | |  |
| **M36** | | Type de mur *(Quel est le matériau principal de votre mur?)*  (1) Brique  (2) Pierre  (3) Béton  (4) tôle  (5) Bois acheté (planche)  (6) Ravinala/falafa  (7) Bambou/volo  (8) Jonc  (9) Terre  (10) Bois ramassé  (11) Autre (à préciser) | | | | | \|__\| murs  ……….. murs_aut | |  |
| **M37** | | Type de toit *(Quel est le matériau principal du toit de votre maison?)*  (1) Béton  (2) Tuile  (3) Tôle  (4) Bois acheté (planche)  (5) Chaume  (6) Ravinala  (7) Autres végétaux  (8) Autre (à préciser) | | | | | \|__\| toit  ……….. toit_aut | |  |
| **M38** | | Type de sol :  (1) Carrelage  (2) Parquet  (3) Ciment  (4) Vinyl (balatum)  (5) Planches en bois achetées  (6) Terre /sable  (7) Végétaux ou bois ramassé (ravinala)  (8) Autre (préciser) | | | | | \|__\| sol  ………. sol_aut | |  |
| **M39** | | Eclairage : *(Quel est le mode d’éclairage habituel ?)*  (1) Electricité  (2) Pétrole lampant  (3) Bougie  (4) Autre (à préciser)  (5) Pas d’éclairage | | | | | \|__\| **eclair**  …………**.eclair_aut** | |  |
| **M40** | | Combustible (cuisine) : *(Quel est le moyen habituel de faire cuire la nourriture tous les jours ?)*  (1) Gaz bouteille  (2) Electricité  (3) Pétrole  (4) Charbon de bois acheté  (5) Bois  (6)Bouses  (7) Autres (à préciser) | | | | | \|__\| combust  ……….combust_aut | |  |
| *Patrimoines* | | | | | | | | |  |
| **M41a**  **M41b**  **M41c**  **M41d**  **M41e**  **M41f**  **M41g**  **M41h**  **M41i**  **M41j**  **M41k**  **M41l**  **M41m**  **M42a**  **M42b**  **M43a**  **M43b**  **M43c**  **M43d**  **M44a**  **M44b**  **M44c**  **M44d**  **M44e**  **M44f**  **M45a**  **M45b**  **M45c**  **M45d**  **M45e**  **M46a**  **M46b**  **M46c**  **M46d**  **M46e**  **M47**  **M47a**  **M47b** | | Le ménage possède-t-il? (noter le nombre)  - Radio  - Télévision  - Ordinateur  - Accès internet  - Téléphone fixe  - Téléphone portable  - Réfrigérateur  - Machine à coudre  - Voiture  - Moto / mobylette  - Camion  - Tracteur  - Autre véhicule à moteur : préciser **Vehm**……………..  - Bicyclette  - Charrette à zébus  - *Animaux domestiques :*  Chiens  Chats  Oiseaux  Autres (à préciser)  - *Animaux de basse-cour :*  Poulets  Oies  Canards  Pintades  Lapins  Autres (à préciser)………………………….  - *Animaux d'élevage :*  Vaches/Zébus  Porcs  Moutons  Chèvres  Autres (à préciser) ………………………..  - *Potagers/Vergers :*  Fruits  Légumes (courgettes, poireaux, chou…)  Tubercules (pommes de terre, patates, igname,…)  Légumineuses (haricots, pois…)  Autres (à préciser)…………………………..  - Rizières  - Rizières en location  - Maisons ou appartements en location | | | | | \|__\|__\| radio  \|__\|__\| tv  \|__\|__\| ordi  \|__\|__\| internet  \|__\|__\| tel_fix  \|__\|__\| tel_mob_nb  \|__\|__\| frigo  \|__\|__\| mach_coud  \|__\|__\| voiture  \|__\|__\| moto  \|__\|__\| camion  \|__\|__\| tract  \|__\|__\| vehic_aut  \|__\|__\| velo  \|__\|__\| charette  \|__\|__\| chien  \|__\|__\| chat  \|__\|__\| oiseau  \|__\|__\| dom_aut_nb  \|__\|__\| poul  \|__\|__\| oie  \|__\|__\| canard  \|__\|__\| pintad  \|__\|__\| lapin  \|__\|__\| basc_aut_nb  \|__\|__\| zebu  \|__\|__\| porc  \|__\|__\| mouton  \|__\|__\| chevre  \|__\|__\| elv_au_nb  \|__\|__\| fruit  \|__\|__\| legume  \|__\|__\| tuberc  \|__\|__\| legumin  \|__\|__\| potag_ aut  \|__\|__\| riziere  \|__\|__\| riziere_loc  \|__\|__\| log_loc | |  |
| *Hygiène du ménage* | | | | | | | | |  |
| **M48** | | Modalité d’évacuation des ordures/déchets ménagers : (*Comment vous débarrassez-vous habituellement de vos ordures ?)*  (1) Collecte de la ville  (2) Dépôt dans une fosse de la ville  (3) Dépôt sur la route  (4) Incinération sur place  (5) Pas de mode d’évacuation fixe  (6) Autre (à préciser) | | | | | \|__\| ordur_evac  ……….. ….ordur_aut | |  |
| **M49** | | Types de latrines et toilettes *(Quel type de toilettes/latrines est principalement utilisé par votre famille ?)*  (1) Toilettes +chasse d’eau, intérieures privées  (2) Toilettes +chasse d’eau, intérieures communes  (3) Latrines creusées extérieures individuelles  (4) Latrines creusées extérieures communes  (5) Dans la nature  (6) Autres (à préciser) | | | | | \|__\| latrine  …………… latrine_aut | |  |
| **M50** | | Local pour se laver (*Où vont le plus souvent les membres de la famille pour se laver ?)*:  (1) Intérieur, pièce spécifique (salle d’eau)  (2) Intérieur sans pièce spécifique (cuisine...)  (3) Extérieur abris spécifique  (4) Extérieur sans abris (dans la cour)  (5) Rivière/ Mare  (6) Autre, préciser | | | | | \|__\| douche  ………… douche_aut | |  |
| **M51** | | Approvisionnement en eau de boisson : *(Où cherchez-vous habituellement l’eau de boisson ?)*  (1) Achetée (eau vive,…)  (2) Robinet privé  (3) Borne fontaine publique  (4) Puits privé  (5) Puits collectif  (6) Forage privé (makiplast…)  (7) Pompe publique d’origine indéterminée  (8) Cours d’eau/ Source  (9) Mare/recueil d’eau de pluie  (10) Autre (à préciser) | | | | | \|__\| eau_bois  …………………….eau_bois_aut | |  |
| **M52** | | Distance entre la maison et le point d’approvisionnement en eau de boisson : (*Combien de temps met-on à pieds pour aller chercher l’eau de boisson ?)*  (1) Moins de 5 minutes  (2) Entre 5 et 10 minutes  (3) Entre 10 et 15 minutes  (4) Entre 15 et 30 minutes  (5) Plus de 30 minutes | | | | | \|__\| eau_dist | |  |
| **M53**  **M53a** | | Modalité de stockage de l’eau de boisson : *(Où stockez-vous habituellement l’eau de boisson ?)*  (1) A l’extérieur (2) A l’intérieur de la maison  (3) Ne stocke pas  Pour ceux qui stockent l’eau de boisson, le contenant de stockage est-il protégé (couvert ou fermé avec un bouchon)? (0) Non (1) Oui | | | | | \|__\| **eau_stock**  \|__\| **Constock** | |  |
| **M54**  **M55** | | Traitement de l’eau de boisson :  *(Faites-vous traiter l’eau de boisson avant de boire ?)*  (1) Oui (0) Non  Si Oui, Quel traitement ?  (1) Filtrer (2) Bouillir (3) Décantation simple  (4) Rajouter des produits (6) Autre (à préciser) | | | | | \|__\| **eau_tt**  \|__\| **Natrait**  ……….**Natrait_aut** | |  |
| **M56** | | Où cuisinez-vous ?  (1) Dans une pièce spécifique à l’intérieur  (2) Dans une pièce commune à l’intérieur  (3) A l’extérieur dans un abri  (4) A l’extérieur dans la cour ou la rue  (5) Autre (à préciser) | | | | | \|__\| cuisine  ……… cuisine_aut | |  |
| *Alimentation du ménage* | | | | | | | | |  |
| **M57** | | Hier, la mère/POHE a-t-elle mangée à la maison ?  (1) Oui (0) Non | | | | | \|__\| **Consmais** | |  |
| **M58** | | Combien avez-vous dépensé en *Ariary* pour la préparation des repas du membre de la famille dans la journée d’hier? (Noter 99999 si le répondant ne sait pas) | | | | | \|__\|__\|__\|__\|__\|  **alim_budg** | |  |
| **M59a**  **M59b**  **M59c**  **M59d**  **M59e** | | Hier, la mère a pris de ?  *Petit déjeuner* : (1) Oui (0) Non  *Goûter/matin* : (1) Oui (0) Non  *Déjeuner* : (1) Oui (0) Non  *Goûter/après-midi* : (1) Oui (0) Non  *Diner* : (1) Oui (0) Non | | | | | \|__\| **Pdejmer**  \|__\| **Gmatmer**  \|__\| **Dejmer**  \|__\| **Gapremer**  \|__\| **Dinmer** | |  |
| *Pratiques alimentaires de la mère/ménage* | | | | | | | | |  |
| Aliments | | | Hier, la mère a consommé ?  (1) Oui (0) Non | Si oui, combien de fois hier? | Est-ce qu’elle a consommé la dernière semaine ?  (1) Oui (0) Non | | | Si oui, combien du jour durant la dernière semaine? (noter de 1 à 7) |  |
| *Céréales* (Riz, blé, mais, sorgho, pâtes alimentaires, pain, biscuits, farines) | | | **M60a** \|__\|  **Cervmer** | **M60b** \|__\|  **Cernbmer** | **M60c** \|__\|  **Cerdsmer** | | | **M60d** \|__\|  **Cernjmer** |  |
| *Racines et tubercules* (Patate douce, taro, manioc, pomme de terre) | | | **M61a** \|__\|  **Rtvmer** | **M61b** \|__\| **Rtnbmer** | **M61c** \|__\| **Rtdsmer** | | | **M61d** \|__\| **Rtnjmer** |  |
| *Légumineuses* (Haricot, Niébé, petits pois, Pois du cap, lentilles,….) | | | **M62a** \|__\|  **Legvmer** | **M62b** \|__\|  **Legnbmer** | **M62c** \|__\|  **Legdsmer** | | | **M62d** \|__\|  **Legnjmer** |  |
| *Lait/produits laitiers* (yaourts, fromage, lait en poudre…) | | | **M63a** \|__\|  **Lvmer** | **M63b** \|__\|  **Lnbmer** | **M63c** \|__\|  **Ldsmer** | | | **M63d** \|__\|  **Lnjmer** |  |
| *Viandes, Volailles*, *Abats* (bœuf, porc, poulet, foie, …) | | | **M64a** \|__\|  **Vivmer** | **M64b** \|__\|  **Vinbmer** | **M64c** \|__\|  **Vidsmer** | | | **M64d** \|__\|  **Vinjmer** |  |
| *Poissons et fruits des mers* (frais ou séché) | | | **M65a** \|__\|  **Povmer** | **M65b** \|__\|  **Ponbmer** | **M65c** \|__\|  **Podsmer** | | | **M65d** \|__\|  **Ponjmer** |  |
| *Œufs* | | | **M66a** \|__\|  **Ovmer** | **M66b** \|__\|  **Onbmer** | **M66c** \|__\|  **Odsmer** | | | **M66d** \|__\|  **Onjmer** |  |
| *Fruits* (Pomme, ananas, banane, avocats, corossol, litchi, Mangue, goyave, papaye, kaki…..) | | | **M67a** \|__\|  **Fruvmer** | **M67b** \|__\|  **Frunbmer** | **M67c** \|__\|  **Frudsmer** | | | **M67d** \|__\|  **Frunjmer** |  |
| *Légumes et brèdes* (Tomate, courgette, haricot vert, chou, carotte, navet, choux de chine,…) | | | **M68a** \|__\|  **Lemvmer** | **M68b** \|__\|  **Lemnbmer** | **M68c** \|__\|  **Lemdsmer** | | | **M68d** \|__\|  **Lemnjmer** |  |
| *Autres* (à préciser)  **Aualimer**  ………………… | | | **M69a** \|__\|  **Alivmer** | **M69b** \|__\|  **Alinbmer** | **M69c** \|__\|  **Alidsmer** | | | **M69d** \|__\|  **Alinjmer** |  |
