# Supplementary material for: The importance of public health, poverty reduction programs and women’s empowerment in the reduction of child stunting in rural areas of Moramanga and Morondava, Madagascar
Source: PLoS One. 2017 Oct 18;12(10):e0186493. doi: 10.1371/journal.pone.0186493 (PMC5646813; doi:10.1371/journal.pone.0186493)
Supplement: S5 Text — (DOCX) [file pone.0186493.s006.docx]

**Questionnaire sur les pratiques alimentaires et de soins des enfants**

| *Identification de l’enfant* | | | | | | | | | | | | | |
| --- | --- | --- | --- | --- | --- | --- | --- | --- | --- | --- | --- | --- | --- |
| **P1a** | Nom et Prénoms de l’enfant : **Nomenf** | | | | | | | | | | | | |
| **P1b** | Numéro d’identification de l’enfant : \|__\|__\|__\|__\|__\|__\|__\|__\|__\|__\|__\|__\|__\| **Idenf** | | | | | | | | | | | | |
| **P2** | Numéro prélèvement **Nprel** | | | | | | | | | | | | |
| **P3** | Sexe de l’enfant :(1) Masculin ; (2) Féminin | | | | | | | | | \|__\| **Senf** | | | |
| **P4a**  **P4b** | Date de naissance de l’enfant :  (1) Vérifiée (2) Déclarée (3) nsp (Si P4b=1 ou 2 →P5) | | | | | | | | | \|__\|__\|/\|__\|__\|/\|__\|__\| **Datenf**  \|__\| **Datenfvd** | | | |
| **P4c** | Age en mois | | | | | | | | | \|__\|__\| **Agenf** | | | |
| **P4d** | L’enfant est-il jumeau ? (1) Oui (0) Non | | | | | | | | | \|__\| **Jumeau** | | | |
| **P5** | Groupe de l’enfant au dépistage (1) MC (2) MA (3) NM | | | | | | | | | \|__\| **Grenfdep** | | | |
| *Mesures anthropométriques* | | | | | | | | | | | | | |
| **Mère ou personne qui s’occupe habituellement l’enfant** | | | | | | | | | | | | | |
| **P6a**  **P6b**  **P6c**  **P6** | *Poids de la mère :*  - Mesure 1  - Mesure 2  - Mesure 3  →Moyenne | | | | | | | | | \|__\|__\|,\|__\|__\| kg **Poidmer 1**  \|__\|__\|,\|__\|__\| kg **Poidmer 2**  \|__\|__\|,\|__\|__\| kg **Poidmer 3**  \|__\|__\|,\|__\|__\| kg **Poidmer** | | | |
| **P7a**  **P7b**  **P7c**  **P7** | *Taille de la mère :*  - Mesure 1  - Mesure2  - Mesure 3  →Moyenne | | | | | | | | | \|__\|__\|__\|,\|__\| cm **Tailmer 1**  \|__\|__\|__\|,\|__\| cm **Tailmer 2**  \|__\|__\|__\|,\|__\| cm **Tailmer 3**  \|__\|__\|__\|,\|__\| cm **Tailmer** | | | |
| **P7d** | La mère présente-t-elle un handicap pouvant affecter  les mesures anthropométriques ? (1) Oui (0) Non | | | | | | | | | \|__\| **Handphymer** | | | |
| **Enfant** | | | | | | | | | | | | | |
| **P8a**  **P8b**  **P8c**  **P8** | *Poids de l’enfant :*  - Mesure 1  - Mesure 2  - Mesure 3  →Moyenne | | | | | | | | | \|__\|__\|,\|__\|__\| kg **Poidenf 1**  \|__\|__\|,\|__\|__\| kg **Poidenf 2**  \|__\|__\|,\|__\|__\| kg **Poidenf 3**  \|__\|__\|,\|__\|__\| kg **Poidenf** | | | |
| **P9a**  **P9b**  **P9c**  **P9** | *Taille de l’enfant :*  - Mesure 1  - Mesure2  - Mesure 3  →Moyenne | | | | | | | | | \|__\|__\|__\|,\|__\| cm **Taillenf 1**  \|__\|__\|__\|,\|__\| cm **Taillenf 2**  \|__\|__\|__\|,\|__\| cm **Taillenf 3**  \|__\|__\|__\|,\|__\| cm **Taillenf** | | | |
| **P10a**  **P10b**  **P10c**  **P10** | *Périmètre brachial de l’enfant :*  - Mesure 1  - Mesure2  - Mesure 3  →Moyenne | | | | | | | | | \|__\|__\|,\|__\| cm **Pbenf 1**  \|__\|__\|,\|__\| cm **Pbenf 1**  \|__\|__\|,\|__\| cm **Pbenf 3**  \|__\|__\|,\|__\| cm **Pbenf** | | | |
| **P11a** | Groupe de l’enfant au moment de l’enquête :  (1) MC (2) MA (3) NM | | | | | | | | | \|__\| **Grenfenq** | | | |
| **P11b** | L’enfant présente-t-il un handicap pouvant affecter  les mesures anthropométriques ? (1) Oui (0) Non | | | | | | | | | \|__\| **Handphyenf** | | | |
| *Pratiques alimentaires de l’enfant* | | | | | | | | | | | | | |
| **P12**  **P12a**  **P12b** | Depuis le dépistage, avez-vous changé le régime alimentaire de cet enfant?  (1) Oui (0) Non  - Si Oui, pourquoi? (1) Mon enfant a grandi en âge (2) En suivant le conseil que vous aviez donné  (3) Autres (à préciser)  - Si oui, qu’est-ce que vous avez changé?  (1) Augmenter la quantité/le nombre du repas (1) Oui (0) Non  (2) Diversifier l’alimentation (1) Oui (0) Non  (3) Introduire de nouveaux aliments (1) Oui (0) Non  - Aliment 1  - Aliment 2  - Aliment 3  (4) Autres (à préciser) | | | | | | | | | \|__\| **Charegenf**  \|__\| **Rchareg**  ……………….. **Rchareg_aut**  \|__\| **Modcha**  \|__\|  \|__\|  ………………..**Alim 1**  ………………..**Alim 2**  ………………..**Alim 3**  ………………..**Modchaut** | | | |
| **P13a**  **P13b** | - Combien de temps après la naissance l’enfant a-t-il été mis au sein pour la première fois ? (1) Moins d’une heure  (2) Moins de 24 heure (3) 24 heure et plus (4) Il n’a jamais été allaité (5) Ne se rappelle pas  - Si 3 ou 4, pourquoi ne l’avez-vous pas allaité au cours de la première journée ? (1) Retard de montée laiteuse (2) Vous pensez que le premier lait n’est pas bon pour le bébé  (3) Vous pensez que le corps du bébé doit se nettoyer d’abord (4) Autres raisons (à préciser) | | | | | | | | | \|__\| **Seinpref**  \|__\|__\| **Rnseinpref**  …………… **Arnseinpref** | | | |
| **P13c**  **P13d** | - Après sa naissance, avez-vous donné autre chose à l’enfant avant de donner le sein ? (1) Oui (0) Non  - Si Oui, que lui avez-vous donné ? (1) du lait maternisé  (2) de l’eau (3) de l’eau sucré (4) du thé/du café  (5) du ranom-bary/du ranon’ampango (6) Autre chose (à préciser) | | | | | | | | | \|__\| **Consavlaim**  \|__\|__\| **Alcons**  …………….**Autalcons** | | | |
| **P14**  **P14a**  **P14b**  **P14c** | Hier, l’enfant a-t-il pris le sein ? (1) Oui ; (0) Non  - Si oui, combien de fois : (1) moins de 5 fois ; (2) de 5 à 9 fois ; (3) de 10 à 14 fois ; (4) 15 fois et plus ; (5) nsp  - Si non, pourquoi ? (1) déjà sevré ; (2) enfant malade ;  (3) Mère séparée de l’enfant / occupée ; (4) Mère malade ; (5) Mère décédée ; (6) Autre (à préciser)  - Si l’enfant a déjà sevré (*Rnsein=1*), quel âge (en mois) avait-il quand vous l’avez sevré ? | | | | | | | | | \|__\| **Prisein**  \|__\| **Freprisein**  \|__\| **Rnsein**  **Rnseinaut**  \|__\|__\| **Agesevra** | | | |
| **P15** | Pour ceux qui allaitent, en quel moment allaitez-vous l’enfant ? (1) à la demande (2) à des moments fixés | | | | | | | | | \|__\| **Momall** | | | |
| **P16** | Hier, l’enfant a-t-il reçu des aliments à l’aide d’un biberon ? (1) Oui ; (0) Non | | | | | | | | | \|__\| **Biberon** | | | |
| Hier, quel type de plat a mangé votre enfant aux différents repas ? | | | | | | | | | | | | | |
|  | | Bouillie **P17a** | | | Snack **P17b** | Fruit **P17c** | | | Plat spécial **P17d** | | | Plat familial **P17e** | |
| *Petit déjeuner* **Pdejenf** | | (1) Oui (0) Non | | | (1) Oui (0) Non | (1) Oui (0) Non | | | (1) Oui (0) Non | | | (1) Oui (0) Non | |
| *Goûter/matin*  **Gmatenf** | | (1) Oui (0) Non | | | (1) Oui (0) Non | (1) Oui (0) Non | | | (1) Oui (0) Non | | | (1) Oui (0) Non | |
| *Déjeuner* **Dejenf** | | (1) Oui (0) Non | | | (1) Oui (0) Non | (1) Oui (0) Non | | | (1) Oui (0) Non | | | (1) Oui (0) Non | |
| *Goûter/aprèsmidi* **Gapremenf** | | (1) Oui (0) Non | | | (1) Oui (0) Non | (1) Oui (0) Non | | | (1) Oui (0) Non | | | (1) Oui (0) Non | |
| *Dîner* **Dinenf** | | (1) Oui (0) Non | | | (1) Oui (0) Non | (1) Oui (0) Non | | | (1) Oui (0) Non | | | (1) Oui (0) Non | |
|  | | | | | | | | | | | | | |
|  | | | | | | | | | | | | | |
| Aliment | | A quel âge en mois a-t-il commencé à consommer de? | | | Hier, combien de fois l’enfant a consommé ?  (Noter 0 si l’enfant n’a pas consommé) | | Quelle quantité l’enfant a-t-il consommée hier?  (en cuillères, bols, verre, pot, nombre, morceau) | | | | | Combien de jour durant la dernière semaine? (Noter 0 si l’enfant n’a pas consommé) | |
| **Céréales** | | | | | | | | | | | | | |
| Ranon’ampango | | **P18a** \|__\|__\|,\|__\| **Rpagenf** | | | **P18b** \|__\| **Rpvenf** | | **Quarpvenf**  \|__\|__\|,\|__\| verre | | | | | **P18c** \|__\| **Rpdsenf** | |
| Bouillie | | **P19a** \|__\|__\|,\|__\| **Bouagenf** | | | **P19b** \|__\| **Bouvenf** | | **Quabouvenf**  \|__\|__\|,\|__\| bol | | | | | **P19c** \|__\| **Boudsenf** | |
| Vary sosoa | | **P20a** \|__\|__\|,\|__\| **Vsagenf** | | | **P20b** \|__\| **Vsvenf** | | **Quavsvenf**  \|__\|__\|,\|__\| bol | | | | | **P20c** \|__\| **Vsdsenf** | |
| Vary maina | | **P21a** \|__\|__\|,\|__\| **Vmagenf** | | | **P21b** \|__\| **Vmvenf** | | **Quavmvenf**  \|__\|__\|,\|__\| bol | | | | | **P21c** \|__\| **Vmdsenf** | |
| Maïs | | **P22a** \|__\|__\|,\|__\| **Maiagenf** | | | **P22b** \|__\| **Maivenf** | | **Quamaivenf**  \|__\|__\|,\|__\| cuillère | | | | | **P22c** \|__\| **Maidsenf** | |
| Pain | | **P23a** \|__\|__\|,\|__\| **Painagenf** | | | **P23b** \|__\| **Painvenf** | | **Quapainvenf**  \|__\|__\|,\|__\| morceau | | | | | **P23c** \|__\| **Paindsenf** | |
| Biscuit | | **P24a** \|__\|__\|,\|__\| **Bisagenf** | | | **P24b** \|__\| **Bisvenf** | | **Quabisvenf**  \|__\|__\|,\|__\| morceau | | | | | **P24c** \|__\| **Bisdsenf** | |
| Pâte | | **P25a** \|__\|__\|,\|__\| **Patagenf** | | | **P25b** \|__\| **Patvenf** | | **Quapatvenf**  \|__\|__\|,\|__\| bol | | | | | **P25c** \|__\| **Patdsenf** | |
| Eau de cuisson de céréales | | **P26a** \|__\|__\|,\|__\| **Bceragenf** | | | **P26b** \|__\| **Bcervenf** | | **Quabcervenf**  \|__\|__\|,\|__\| verre | | | | | **P26c** \|__\| **Bcerdsenf** | |
| Autres céréales  **Atcerenf** | | **P27a** \|__\|__\|,\|__\|  **Atceragenf** | | | **P27b** \|__\| **Atcervenf** | | **Quatcervenf**  \|__\|__\|,\|__\| | | | | | **P27c** \|__\| **Atcerdsenf** | |
| **Racines et tubercules** | | | | | | | | | | | | | |
| Manioc | | **P28a** \|__\|__\|,\|__\| **Managenf** | | | **P28b** \|__\| **Manvenf** | | **Quamanvenf**  \|__\|__\|,\|__\| morceau | | | | | **P28c** \|__\| **Mandsenf** | |
| Patate douce | | **P29a** \|__\|__\|,\|__\| **Pagenf** | | | **P29b** \|__\| **Pavenf** | | **Quapavenf**  \|__\|__\|,\|__\| morceau | | | | | **P29c** \|__\| **Padsenf** | |
| Taro | | **P30a** \|__\|__\|,\|__\| **Tagenf** | | | **P30b** \|__\| **Tavenf** | | **Quatavenf**  \|__\|__\|,\|__\| morceau | | | | | **P30c** \|__\| **Tadsenf** | |
| Pomme de terre | | **P31a** \|__\|__\|,\|__\| **Potagenf** | | | **P31b** \|__\| **Potvenf** | | **Quapotvenf**  \|__\|__\|,\|__\| morceau | | | | | **P31c** \|__\| **Potdsenf** | |
| Eau de cuisson de racines/  tubercules | | **P32a** \|__\|__\|,\|__\| **Brtagenf** | | | **P32b** \|__\| **Brtvenf** | | **Quabrtvenf**  \|__\|__\|,\|__\| verre | | | | | **P32c** \|__\| **Brtdsenf** | |
| Autres racines/tubercules **Aurtenf** | | **P33a**\|__\|__\|,\|__\|  **Aurtagenf** | | | **P33b** \|__\| **Aurtvenf** | | **Quaurtvenf**  \|__\|__\|,\|__\| morceau | | | | | **P33c** \|__\| **Aurtdsenf** | |
| **Légumineuses** | | | | | | | | | | | | | |
| Haricot | | **P34a** \|__\|__\|,\|__\| **Haragenf** | | | **P34b** \|__\| **Harvenf** | | **Quaharvenf**  \|__\|__\|,\|__\| cuillère | | | | | **P34c** \|__\| **Hardsenf** | |
| Ambérique | | **P35a** \|__\|__\|,\|__\|  **Ambagenf** | | | **P35b** \|__\| **Ambvenf** | | **Quambvenf**  \|__\|__\|,\|__\| cuillère | | | | | **P35c** \|__\| **Ambdsenf** | |
| Pois du cap | | **P36a** \|__\|__\|,\|__\| **Pcagenf** | | | **P36b** \|__\| **Pcvenf** | | **Quapcvenf**  \|__\|__\|,\|__\| cuillère | | | | | **P36c** \|__\| **Pcdsenf** | |
| Niébé | | **P37a** \|__\|__\|,\|__\| **Niagenf** | | | **P37b** \|__\| **Nivenf** | | **Quanivenf**  \|__\|__\|,\|__\| cuillère | | | | | **P37c** \|__\| **Nidsenf** | |
| Lentille | | **P38a** \|__\|__\|,\|__\| **Lenagenf** | | | **P38b** \|__\| **Lenvenf** | | **Qualenvenf**  \|__\|__\|,\|__\| cuillère | | | | | **P38c** \|__\| **Ledsenf** | |
| Voandzou | | **P39a** \|__\|__\|,\|__\| **Voagenf** | | | **P39b** \|__\| **Vovenf** | | **Quavovenf**  \|__\|__\|,\|__\| cuillère | | | | | **P39c** \|__\| **Vodsenf** | |
| Arachide | | **P40a** \|__\|__\|,\|__\| **Aragenf** | | | **P40b** \|__\| **Arvenf** | | **Quarvenf**  \|__\|__\|,\|__\| cuillère | | | | | **P40c** \|__\| **Ardsenf** | |
| Eau de cuisson de légumineuses | | **P41a** \|__\|__\|,\|__\| **Blegagenf** | | | **P41b** \|__\| **Blegvenf** | | **Quablegvenf**  \|__\|__\|,\|__\| verre | | | | | **P41c** \|__\| **Blegdsenf** | |
| Autres légumineuses  **Aulegenf** | | **P42a**\|__\|__\|,\|__\| **Aulegagenf** | | | **P42b** \|__\| **Aulegvenf** | | **Quaulegvenf**  \|__\|__\|,\|__\| cuillère | | | | | **P42c** \|__\| **Aulegdsenf** | |
| **Lait et produits laitiers** | | | | | | | | | | | | | |
| Lait autre que maternel | | **P43a** \|__\|__\|,\|__\| **Laiagenf** | | | **P43b** \|__\| **Laivenf** | | **Qualaivenf**  \|__\|__\|,\|__\| verre | | | | | **P43c** \|__\| **Laidsenf** | |
| Yaourt | | **P44a** \|__\|__\|,\|__\| **Yoagenf** | | | **P44b** \|__\| **Yovenf** | | **Quayovenf**  \|__\|__\|,\|__\| pot | | | | | **P44c** \|__\| **Yodsenf** | |
| Autres produits laitiers **Aulenf** | | **P45a** \|__\|__\|,\|__\| **Aulagenf** | | | **P45b** \|__\| **Aulvenf** | | **Quaulvenf**  \|__\|__\|,\|__\| | | | | | **P45c** \|__\| **Auldsenf** | |
| **Viande, Volaille, poisson et ses dérivés** | | | | | | | | | | | | | |
| Bœuf /dérivés | | **P46a** \|__\|__\|,\|__\| **Boagenf** | | | **P46b** \|__\| **Bovenf** | | **Quabovenf**  \|__\|__\|,\|__\| morceau | | | | | **P46c** \|__\| **Bodsenf** | |
| Porc/dérivés | | **P47a** \|__\|__\|,\|__\| **Poagenf** | | | **P47b** \|__\| **Povenf** | | **Quapovenf**  \|__\|__\|,\|__\| morceau | | | | | **P47c** \|__\| **Podsenf** | |
| Saucisse | | \|__\|__\|,\|__\| **Sauagenf** | | | \|__\| **Sauvenf** | | **Quasauvenf**  \|__\|__\|,\|__\| morceau | | | | | \|__\| **Saudsenf** | |
| Volaille | | **P48a** \|__\|__\|,\|__\| **Volagenf** | | | **P48b** \|__\| **Volvenf** | | **Quavolvenf**  \|__\|__\|,\|__\| morceau | | | | | **P48c** \|__\| **Voldsenf** | |
| Abats | | **P49a** \|__\|__\|,\|__\| **Abagenf** | | | **P49b** \|__\| **Abvenf** | | **Quabvenf**  \|__\|__\|,\|__\| morceau | | | | | **P49c** \|__\| **Abdsenf** | |
| Poisson frais | | **P50a** \|__\|__\|,\|__\| **Pfagenf** | | | **P50b** \|__\| **Pfvenf** | | **Quapfvenf**  \|__\|__\|,\|__\| morceau | | | | | **P50c** \|__\| **Pfdsenf** | |
| Poisson séché | | **P51a** \|__\|__\|,\|__\| **Psagenf** | | | **P51b** \|__\| **Psvenf** | | **Quapsvenf**  \|__\|__\| morceau | | | | | **P51c** \|__\| **Psdsenf** | |
| Crevettes | | **P52a** \|__\|__\|,\|__\| **Cragenf** | | | **P52b** \|__\| **Crvenf** | | **Quacrvenf**  \|__\|__\|,\|__\| morceau | | | | | **P52c** \|__\| **Crdsenf** | |
| Bouillon de viande/volaille/ poisson… | | **P53a** \|__\|__\|,\|__\| **Bviagenf** | | | **P53b** \|__\| **Bvivenf** | | **Quabvivenf**  \|__\|__\|,\|__\| verre | | | | | **P53c** \|__\| **Bvidsenf** | |
| Autre produits mer/eau douce  **Pmenf** | | **P54a** \|__\|__\|,\|__\| **Pmagenf** | | | **P54b** \|__\| **Pmvenf** | | **Quapmvenf**  \|__\|__\|,\|__\| | | | | | **P54c** \|__\| **Pmdsenf** | |
| **Œuf** | | **P55a** \|__\|__\|,\|__\| **Oagenf** | | | **P55b** \|__\| **Ovenf** | | **Quaovenf**  \|__\|__\|,\|__\| pièces | | | | | **P55c** \|__\| **Odsenf** | |
| **Légumes et brèdes** | | | | | | | | | | | | | |
| Carotte | | **P56a** \|__\|__\|,\|__\| **Caagenf** | | | **P56b** \|__\| **Cavenf** | | **Quacavenf**  \|__\|__\|,\|__\| morceau/cuillère | | | | | **P56c** \|__\| **Cadsenf** | |
| Chou | | **P57a** \|__\|__\|,\|__\| **Choagenf** | | | **P57b** \|__\| **Chovenf** | | **Quachovenf**  \|__\|__\|,\|__\| morceau/cuillère | | | | | **P57c** \|__\| **Chodsenf** | |
| Citrouille | | **P58a** \|__\|__\|,\|__\| **Ciagenf** | | | **P58b** \|__\| **Civenf** | | **Quacivenf**  \|__\|__\|,\|__\|morceau/cuillère | | | | | **P58c** \|__\| **Cidsenf** | |
| Courgette | | **P59a** \|__\|__\|,\|__\| **Coagenf** | | | **P59b** \|__\| **Covenf** | | **Quacovenf**  \|__\|__\|,\|__\| morceau/cuillère | | | | | **P59c** \|__\| **Codsenf** | |
| Haricot vert | | **P60a** \|__\|__\|,\|__\| **Hvagenf** | | | **P60b** \|__\| **Hvvenf** | | **Quahvvenf**  \|__\|__\|,\|__\| morceau/cuillère | | | | | **P60c** \|__\| **Hvdsenf** | |
| Oignons | | **P61a** \|__\|__\|,\|__\| **Oiagenf** | | | **P61b** \|__\| **Oivenf** | | **Quaoivenf**  \|__\|__\|,\|__\| morceau | | | | | **P61c** \|__\| **Oidsenf** | |
| Tomate | | **P62a** \|__\|__\|,\|__\| **Toagenf** | | | **P62b** \|__\| **Tovenf** | | **Quatovenf**  \|__\|__\|,\|__\| morceau | | | | | **P62c** \|__\| **Todsenf** | |
| Autres légumes **Lemenf** | | **P63a** \|__\|__\|,\|__\|  **Lemagenf** | | | **P63b** \|__\| **Lemvenf** | | **Qualemvenf**  \|__\|__\|,\|__\| morceau/cuillère | | | | | **P63c** \|__\| **Lemdsenf** | |
| Choux de chine | | **P64a** \|__\|__\|,\|__\| **Ccagenf** | | | **P64b** \|__\| **Ccvenf** | | **Quaccvenf**  \|__\|__\|,\|__\| cuillère | | | | | **P64c** \|__\| **Ccdsenf** | |
| Anamamy | | **P65a** \|__\|__\|,\|__\| **Amagenf** | | | **P65b** \|__\| **Amvenf** | | **Quamvenf**  \|__\|__\|,\|__\| cuillère | | | | | **P65c** \|__\| **Amdsenf** | |
| Anandrano | | **P66a** \|__\|__\|,\|__\| **Anagenf** | | | **P66b** \|__\| **Anvenf** | | **Quanvenf**  \|__\|__\|,\|__\| cuillère | | | | | **P66c** \|__\| **Andsenf** | |
| Anatsinahy | | **P67a** \|__\|__\|,\|__\| **Atagenf** | | | **P67b** \|__\| **Atvenf** | | **Quatvenf**  \|__\|__\|,\|__\| cuillère | | | | | **P67c** \|__\| **Atdsenf** | |
| Ravim-bomanga | | **P68a** \|__\|__\|,\|__\| **Rmagenf** | | | **P68b** \|__\| **Rmvenf** | | **Quarmvenf**  \|__\|__\|,\|__\| cuillère | | | | | **P68c** \|__\| **Rmdsenf** | |
| Ravin-tsaosety | | **P69a** \|__\|__\|,\|__\| **Rsagenf** | | | **P69b** \|__\| **Rsvenf** | | **Quarsvenf**  \|__\|__\|,\|__\| cuillère | | | | | **P69c** \|__\| **Rsdsenf** | |
| Ravitoto | | **P70a** \|__\|__\|,\|__\| **Rtagenf** | | | **P70b** \|__\| **Rtvenf** | | **Quartvenf**  \|__\|__\|,\|__\| cuillère | | | | | **P70c** \|__\| **Rtdsenf** | |
| Bouillon de légumes/brèdes | | **P71a** \|__\|__\|,\|__\| **Blemagenf** | | | **P71b** \|__\| **Blemvenf** | | **Quablemvenf**  \|__\|__\|,\|__\| verre | | | | | **P71c** \|__\| **Blemdsenf** | |
| Autres brèdes **Brenf** | | **P72a** \|__\|__\|,\|__\| **Bragenf** | | | **P72b** \|__\| **Brvenf** | | **Quabrvenf**  \|__\|__\|,\|__\| cuillère | | | | | **P72c** \|__\| **Brdsenf** | |
| **Fruits** | | | | | | | | | | | | | |
| Jus de fruit | | **P73a** \|__\|__\|,\|__\| **Jusagenf** | | | **P73b** \|__\| **Jusvenf** | | **Quajusvenf**  \|__\|__\|,\|__\| verre | | | | | **P73c** \|__\| **Jusdsenf** | |
| Agrumes | | **P74a** \|__\|__\|,\|__\| **Agragenf** | | | **P74b** \|__\| **Agrvenf** | | **Quagrvenf**  \|__\|__\|,\|__\| pièces | | | | | **P74c** \|__\| **Agrdsenf** | |
| Banane | | **P75a** \|__\|__\|,\|__\| **Banagenf** | | | **P75b** \|__\| **Banvenf** | | **Quabanvenf**  \|__\|__\| ,\|__\|pièces | | | | | **P75c** \|__\| **Bandsenf** | |
| Canne à sucre | | **P76a** \|__\|__\|,\|__\| **Canagenf** | | | **P76b** \|__\| **Canvenf** | | **Quacanvenf**  \|__\|__\|,\|__\| pièces | | | | | **P76c** \|__\| **Candsenf** | |
| Corossol | | **P77a** \|__\|__\|,\|__\| **Coragenf** | | | **P77b** \|__\| **Corvenf** | | **Quacorvenf**  \|__\|__\|,\|__\| pièces | | | | | **P77c** \|__\| **Cordsenf** | |
| Goyave | | **P78a** \|__\|__\|,\|__\| **Goagenf** | | | **P78b** \|__\| **Govenf** | | **Quagovenf**  \|__\|__\|,\|__\| pièces | | | | | **P78c** \|__\| **Godsenf** | |
| Mangue | | **P79a** \|__\|__\|,\|__\| **Magagenf** | | | **P79b** \|__\| **Magvenf** | | **Quamagvenf**  \|__\|__\|,\|__\| pièces | | | | | **P79c**\|__\| **Magdsenf** | |
| Papaye | | **P80a** \|__\|__\|,\|__\| **Papagenf** | | | **P80b** \|__\| **Papvenf** | | **Quapapvenf**  \|__\|__\|,\|__\| pièces | | | | | **P80c** \|__\| **Papdsenf** | |
| Autres fruits **Fruitenf** | | **P81a** \|__\|__\|,\|__\| **Fruagenf** | | | **P81b** \|__\| **Fruvenf** | | **Quafruvenf**  \|__\|__\|,\|__\| pièces | | | | | **P81c** \|__\| **Frudsenf** | |
| **Huile et graisse** | | | | | | | | | | | | | |
| Huile végétale | | **P82a** \|__\|__\|,\|__\| **Huvagenf** | | | **P82b** \|__\| **Huvenf** | | | | | | | **P82c** \|__\| **Huvdsenf** | |
|  |  | - Si l’enfant a consommé de plat rajouté d’huile végétale hier, combien de personne a consommé ce repas ?  - Quantité d’huile rajoutée dans le repas de la famille hier **Quahuv**  - Quantité du plat consommé par l’enfant **Platvenf** | | | | | | | | | | \|__\|__\|  \|__\|__\| cuillère  \|__\|__\| bol | |
| Graisse animale | | **P83a** \|__\|__\|,\|__\| **Hanagenf** | | | **P83b** \|__\| **Hanvenf** | | **Quahanvenf**  \|__\|__\|,\|__\| cuillère | | | | | **P83c** \|__\| **Handsenf** | |
| Autres (à préciser)  **Authugr** | | **P84a** \|__\|__\|,\|__\| **Hugragenf** | | | **P84b** \|__\| **Hugrvenf** | | **Quahugrvenf**  \|__\|__\|,\|__\| cuillère | | | | | **P84c** \|__\| **Hugrdsenf** | |
| **Produits sucrés** | | | | | | | | | | | | | |
| Eau sucré | | **P85a** \|__\|__\|,\|__\| **Esagenf** | | | **P85b** \|__\| **Esvenf** | | **Quaesvenf**  \|__\|__\|,\|__\| verre | | | | | **P85c** \|__\| **Esdsenf** | |
| Thé, café | | **P86a** \|__\|__\|,\|__\| **Tcagenf** | | | **P86b** \|__\| **Tcvenf** | | **Quacvenf**  \|__\|__\|,\|__\| verre | | | | | **P86c** \|__\| **Tcdsenf** | |
| Miel | | **P87a** \|__\|__\|,\|__\| **Miagenf** | | | **P87b** \|__\| **Mivenf** | | **Quamivenf**  \|__\|__\|,\|__\|cuillère | | | | | **P87c** \|__\| **Midsenf** | |
| Bonbon | | **P88a** \|__\|__\|,\|__\| **Bonagenf** | | | **P88b** \|__\| **Bonvenf** | | **Quabonvenf**  \|__\|__\|,\|__\| pièce | | | | | **P88c** \|__\| **Bondsenf** | |
| **Eau** | | **P89a** \|__\|__\|,\|__\| **Eagenf** | | | **P89b** \|__\| **Eavenf** | | **Quaeavenf**  \|__\|__\|,\|__\| verre | | | | | **P89c** \|__\| **Eadsenf** | |
| Autres aliments **Aualim**  ___________ | | **P90a** \|__\|__\|,\|__\| **Aliagenf** | | | **P90b** \|__\| **Alivenf** | | **Qualivenf**  \|__\|__\|,\|__\| | | | | | **P90c** \|__\| **Alidsenf** | |
| *Pratiques sanitaires* | | | | | | | | | | | | | |
| **P91** | L’enfant possède-t-il un carnet de santé, de pesée ou de vaccination ? (1) Oui ; (0) Non ; (2) Nsp | | | | | | | | | | \|__\| **Carnet** | | |
| **P92a**  **P92b** | Quel est son poids à la naissance (en kg) ?  (1) Vérifié; (2) Déclaré; (3) Inconnu | | | | | | | | | | \|__\|__\|, \|__\|__\| kg  **Pnaiss**  \|__\| **Pverd** | | |
| **P93**  **P93a**  **P93b** | Pour cet enfant, avez-vous suivi des consultations prénatales?  (1) Oui ; (0) Non ; (2) Nsp  Si oui, combien de fois ? (Noter 99 si « ne sait pas »)  - Chez le médecin ou sage-femme  - Chez la matrone | | | | | | | | | | \|__\| **Conspren**  \|__\|__\|  **Consaf**  \|__\|__\|  **Conmat** | | |
| **P94** | Où a eu lieu l’accouchement ? (1) hôpital/CSB, (2) domicile (3) Autre (à préciser) | | | | | | | | | | \|__\| **Lieuac**  ……………………**Lieuac_aut** | | |
| **P95a**  **P95b** | Dans les 3 derniers mois, avez-vous emmené l’enfant dans un centre de santé ? (1) Oui (0) Non  Si oui, pourquoi ?  (1) Vaccination (2) Pesée (3) Maladie  (4) Autre (à préciser) | | | | | | | | | | \|__\| **Csant**  \|__\| **Rcsant**  …………………… **Aursant** | | |
| **P96a**  **P96b** | Est-ce que l’enfant a-t-il déjà reçu une dose de vitamine A comme cela dans les 6 derniers mois?  *(Montrez les types d’ampoules / capsule / sirops les plus communs)*  (1) Oui ; (0) Non ; (2) Non concerné (3) Nsp  Si oui, date de dernière prise de vitamine A | | | | | | | | | | \|__\| **Convita**  \|__\|__\|/\|__\|__\|/\|__\|__\| **Datvita** | | |
| **P97a**  **P97b** | Au cour des 6 derniers mois, est ce que l’enfant a pris des médicaments contre les vers intestinaux ?  (1) Oui ; (0) Non ; (2) Non concerné (3) Nsp  Si oui, date de dernière prise de médicament contre les vers intestinaux | | | | | | | | | | \|__\| **Medver**  \|__\|__\|/\|__\|__\|/\|__\|__\| **Datmed** | | |
| **P98**  **P98a**  **P98b**  **P98c**  **P98d** | L’enfant a-t-il été pris en charge pour avoir une aide alimentaire?  (1) Oui ; (0) Non ; (2) Nsp  Si Oui, pourquoi ? :  (1) Malnutrition (2) Pauvreté (3) Autres (à préciser)  - Quel (s) type(s) d’aliments avez-vous reçus?  (1) Farine infantile/bouillie (2) lait en poudre  (3) Autre (à préciser)  - Date du début de l’aide alimentaire  - Date de fin de l’aide alimentaire | | | | | | | | | | \|__\| **Aidalim**  \|__\| **Raidalim**  \|__\| **Talirec**  ……………………… **Aualirec**  \|__\|__\|/\|__\|__\|/\|__\|__\| **Datdeb**  \|__\|__\|/\|__\|__\|/\|__\|__\| **Datfin** | | |
| **P99**  **P99a**  **P99b**  **P99c**  **P99d** | L’enfant a-t-il été pris en charge dans un centre de récupération nutritionnelle?  (1) Oui ; (0) Non ; (2) Nsp  Si Oui, pourquoi ? (1) Atteint de la malnutrition  (2) Autres (à préciser)  - Quel (s) type(s) d’aliments avez-vous reçus?  (1) Plumpy nut/immunit  (2) Autre (à préciser)  - Date de début de prise en charge  - Date de fin de prise en charge | | | | | | | | | | \|__\| **Pricren**  \|__\| **Rpricren**  \|__\| **Alimrec**  …………………...**Aualimrec**  \|__\|__\|/\|__\|__\|/\|__\|__\| **Datdpr**  \|__\|__\|/\|__\|__\|/\|__\|__\| **Datfpr** | | |
| *Les vaccins suivants ont-ils été effectués ?* | | | | | | | | | | | | | |
| Vaccin | | | | (1) Oui (0) Non | | | | | (1) Vérifié; (2) Déclaré | | | | |
| BCG | | | | **P100a** \|__\| **Vbcg** | | | | | **P100b**  \|__\| **Bcgvd** | | | | |
| Polio O | | | | **P101a** \|__\| **Vpolioa** | | | | | **P101b** \|__\| **Polioavd** | | | | |
| DTCHB 1 | | | | **P102a** \|__\| **Dtchba** | | | | | **P102b** \|__\| **Dtchbavd** | | | | |
| Polio 1 | | | | **P103a** \|__\| **Poliob** | | | | | **P103b** \|__\| **Poliobvd** | | | | |
| PCV10 1 | | | | **P104a** \|__\| **Pcva** | | | | | **P104b** \|__\| **Pcvavd** | | | | |
| DTCHB 2 | | | | **P105a**  \|__\| **Dtchbb** | | | | | **P105b** \|__\| **Dtchbbvd** | | | | |
| Polio 2 | | | | **P106a** \|__\| **Polioc** | | | | | **P106b** \|__\| **Poliocvd** | | | | |
| PCV10 2 | | | | **P107a** \|__\| **Pcvb** | | | | | **P107b** \|__\| **Pcvbvd** | | | | |
| DTCHB 3 | | | | **P108a**  \|__\| **Dtchbc** | | | | | **P108b** \|__\| **Dtchbcvd** | | | | |
| Polio 3 | | | | **P109a** \|__\| **Poliod** | | | | | **P109b** \|__\| **Poliodvd** | | | | |
| PCV10 3 | | | | **P110a**  \|__\| **Pcvc** | | | | | **P110b** \|__\| **Pcvcvd** | | | | |
| VAR | | | | **P111a**  \|__\| **Var** | | | | | **P111b** \|__\| **Varvd** | | | | |
| Vaccin anti-rotavirus :  Dose 1  Dose 2 | | | | **P112a** \|__\| **Vrotvirus 1**  **P113a** \|__\| **Vrotvirus 2** | | | | | **P112b** \|__\| **Vrotvirusvd 1**  **P113b** \|__\| **Vrotvirusvd 2** | | | | |
| *Symptômes présentés par l’enfant* | | | | | | | | | | | | | |
| Liste des symptômes | | | | | Présence des symptômes au moment de l’enquête  (1) Oui (0) Non | | | | Maladies ou symptômes survenues durant les 14 jours précédant l’enquête (1) Oui (0) Non | | | | |
| Fièvre | | | | | **P114a** \|__\| **Fievreme** | | | | **P114b** \|__\| **Fievrede** | | | | |
| Toux | | | | | **P115a**  \|__\| **Touxme** | | | | **P115b** \|__\| **Touxde** | | | | |
| Anorexie | | | | | **P116a** \|__\| **Anorexme** | | | | **P116b** \|__\| **Anorexde** | | | | |
| Ecoulement nasal | | | | | **P117a** \|__\| **Ecnasme** | | | | **P117b** \|__\| **Ecnasde** | | | | |
| Vomissement | | | | | **P118a** \|__\| **Vomime** | | | | **P118b** \|__\| **Vomide** | | | | |
| Douleur abdominale | | | | | **P119a**  \|__\| **Dolabme** | | | | **P119b** \|__\| **Dolabde** | | | | |
| Diarrhée | | | | | **P120a** \|__\| **Diarrheme** | | | | **P120b** \|__\| **Diarrhede** | | | | |
| En cas de diarrhées  (**P120a ou P120b =1**):  - Nombre de selles émises par l’enfant pour une journée ?  - Consistance? :  - (1) Molles  - (2) Liquides  - (3) Pâteuses | | | | | **P120c** \|__\| **Nbselleme**  **P120d** \|__\| **Consistme** | | | | **P120e** \|__\| **Nbsellede**  **P120f** \|__\| **Consistde** | | | | |
| Conjonctivite | | | | | **P121a** \|__\| **Conjome** | | | | **P121b** \|__\| **Conjode** | | | | |
| Otalgie | | | | | **P122a** \|__\| **Otalgime** | | | | **P122b** \|__\| **Otalgide** | | | | |
| Dyspnée | | | | | **P123a** \|__\| **Dyspneme** | | | | **P123b** \|__\| **Dyspnede** | | | | |
| Eruptions cutanées | | | | | **P124a** \|__\| **Ercutme** | | | | **P124b** \|__\| **Ercutde** | | | | |
| Autres maladies  **Autmala** | | | | | **P125a** \|__\| **Autmalme** | | | | **P125b** \|__\| **Autmalde** | | | | |
| L’enfant a pris de(s)médicaments ?  (1) Oui (0) Non  Si oui, quel(s) médicaments a-t-il pris ?  - Medicament 1:  - Medicament 2:  - Medicament 3:  - Medicament 4:  - Medicament 5:  - Medicament 6 : | | | | | **P126a** \|__\| **Medprisme**  ……………. **Medme 1**  ……………. **Medme 2**  ……………. **Medme 3**  ……………. **Medme 4**  …………….. **Medme 5**  …………….. **Medme 6** | | | | **P126b** \|__\| **Medprisde**  ……………. **Medde 1**  ……………. **Medde 2**  ……………. **Medde 3**  ……………. **Medde 4**  …………….. **Medde 5**  …………….. **Medde 6** | | | | |
